# Supplementary material for: Akwenda intervention programme for children and youth with cerebral palsy in a low-resource setting in sub-Saharan Africa: protocol for a quasi-randomised controlled study
Source: BMJ Open. 2021 Mar 8;11(3):e047634. doi: 10.1136/bmjopen-2020-047634 (PMC7942240; doi:10.1136/bmjopen-2020-047634)
Supplement: Supplementary data [file bmjopen-2020-047634supp001.pdf]

Supplement 1:

The Akwenda cerebral palsy intervention programme

The Akwenda cerebral palsy (CP) programme was developed by an international team of experts within childhood disabilities and rehabilitation, an academic team from Uganda, a team from Malamulele Onward in South Africa, a team of therapists and a social worker working in Eastern Uganda, as well as four caregivers from the study area. Findings from our previous studies in the same cohort identifying the needs among the children and families, and the gaps in service provision also informed the process. The first step identified key principles based on current concepts of disability, rehabilitation and interventions for children with CP which would be possible to implement in low-resource settings. This involved reviewing literature and learning from programmes developed for low-resource settings with no or few health professionals. From this review,, the parent-led Carer-2-Carer Training Programme developed and implemented by Malamulele Onward in Johannesburg, South Africa was chosen to form the core of the Akwenda programme.[1] The Malamulele Onward Carer-2-Carer programme was chosen because it targeted children with CP over the age of two years; it had been successfully used in Southern African settings for a number of years; and it was specifically designed to be delivered by parents with low literacy levels.[2] The second step involved clarifying the purpose of and agreeing on goals for the intervention programme. Based on the principles and goals formulated in previous steps, the five components of the intervention were defined in the third step.

Step 1: Key principles

The ICF framework

The first principle is the International Classification of Functioning and Disability and Health (ICF) framework which defines disability not as a characteristic of a person but as the interaction between health conditions and environmental and personal factors at the level of: i) body structure and function; ii) activity, and iii) participation in the family and society. The ICF has had an immense influence on changing perspectives of disability over the last decades in shifting the primary focus of interventions from impairments to participation and inclusion whilst emphasizing the need to address barriers, e.g., attitudinal, institutional and environmental.[3] The ICF framework has enabled community-based services to be brought closer to the children. It has also facilitated family oriented services, i.e. services that directly target the family's needs for information, education and psychosocial support.[4] The Akwenda CP programme has adopted the ICFs holistic view on disability whereby activity and participation are the main focuses of the programme. The programme also embraces the

community-based and family oriented concept, through utilizing trained caregivers to train other caregivers, and by moving the services into the communities and homes of the participants.

Evidence based best practice

The second principle was to ensure that the Akwenda CP programme was based on evidence based best practice. A plethora of interventions are used today for children with CP, many without supportive scientific data.[5] The past decades have seen a surge of clinical trials resulting in a wider range of evidence based interventions, as well as interventions with strong evidence that they are not effective.[5] This emerging evidence shows that interventions improving functional ability in children with CP are child-active approaches where the child is actively practicing real-life tasks. Such child-active approaches are supported by current neuroscience demonstrating activity dependent learning induced plasticity in the brain underlying the learning and improvement of motor skills.[6] With little available information on evidence-based practices from LMIC, the activities of the Akwenda programme are therefore based on best clinical practice derived from clinical studies in HIC, adapted to the low income context in this rural part of Uganda. We have in addition also sought inspiration from programmes and studies done in LMIC which provide innovative strategies to provide the best care available given limited resources. [7 8]

Universal health coverage and universal access

The third principle is based on the WHO's goal of universal health coverage and universal access, which aims to ensure that all people obtain the health and rehabilitation services they need without fear of financial hardship.[9] To achieve universal health and rehabilitation coverage, services should be physically accessible, affordable and acceptable to patients. This means that services need to be available; located close to people; and should not entail financial hardship. Not only the expense of the health services but also the indirect and opportunity costs, e.g. transportation and time away from work need to be taken into account. Acceptability captures people's willingness to seek services, and depends on how patients perceive services and how social and cultural attitudes of the health provider encourage or discourage them from seeking services.[9] We have tried to ensure that the Akwenda CP programme is physically accessible, affordable and acceptable. Physical accessibility and affordability will be ensured through locating the activities in the community, utilising locally available resources for technical assistive devices, optimising the limited therapist time, and the utilization of trained caregivers. The programme also includes a communication and advocacy component aimed to ensure acceptability.

Step 2: Goals

Three general goals based on the principles and the needs of children and youth with cerebral palsy and their families, which had been identified in previous studies[10-12] were agreed upon:

- To improve motor function, activity and participation of the children and youth with cerebral palsy
- To increase knowledge and skill of the caregivers, reduce their stress and improve their mental health

- To facilitate children's inclusion and participation in society through increasing public awareness and reducing stigma in the community.

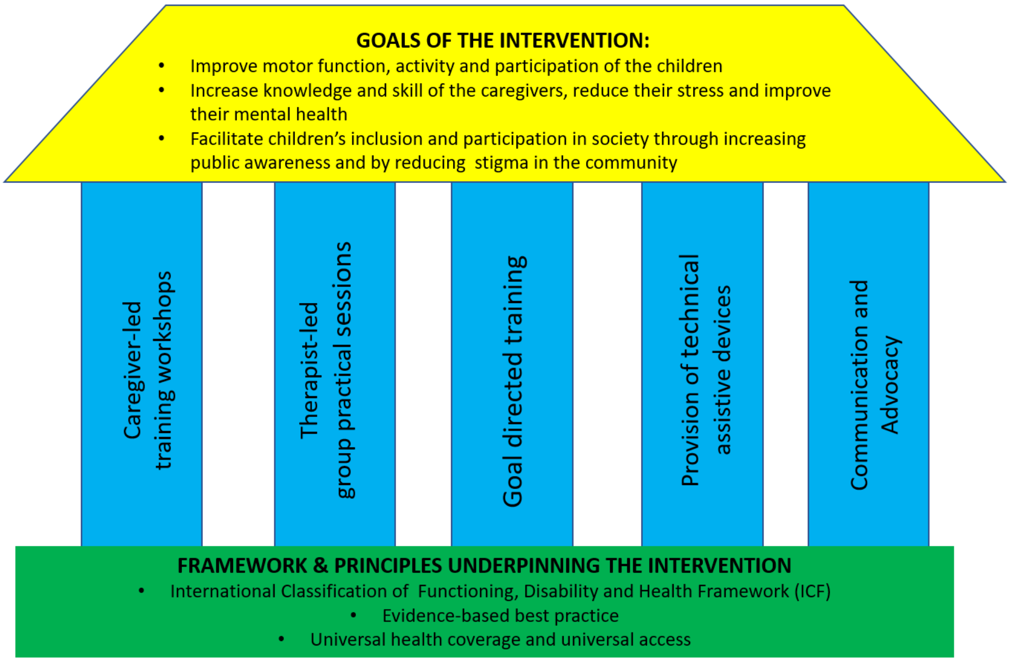

Figure 1: Illustration of the Akwenda CP programme: based on three key principles; comprising five intervention components; and aiming to achieve three goals.

Step 3: Five intervention components

Based on the principles and goals formulated in previous steps, the five components of the Akwenda CP programme are (See Figure1):

1. Caregiver-led training workshops
2. Therapist-led practical group sessions and workshops
3. Provision of technical assistive devices (TAD)
4. Goal directed trainng
5. Communication and advocacy for behavioural and social change (C&A)

Component 1: Caregiver-led training workshops

The caregiver-led training workshops of the Malamulele Onward Carer-2-Carer Training Programme (MOC2CTP) form the cores of the Akwenda CP programme.[1 2] The MOC2CTP trains primary caregivers of children with CP to be Parent Facilitators, able to organise and lead seven training workshops (Table 1). The MOC2CTP was developed and refined over a seven-year period by a multidisciplinary team of three therapists. It differs from the ABAaNA

parent-led early intervention programme in that it (i) was intentionally designed to be run by parents with low literacy levels, hence it uses very simple language and a simple layout with step-by-step instructions; (ii) was designed to be run by parents independent of a therapist being present; (iii) uses a problem solving approach based on functional classification and the main clinical subtypes and (iv) the target group is children over the age of two years. The original group of 20 Parent Facilitators themselves were major contributors to the final product through their feedback and experiences in running the programme.

	**Title of workshop**	**Outcomes**
1	What is CP and how does it affect my child?	• Be able to explain what CP is to a family member<br>• Know the GMFCS Level the child falls into<br>• Know what kind (subtype) of CP the child has<br>• Understand the possible causes of CP, and what does not cause CP, especially that CP is not a disease and not her fault
2	CP as a way of life	• What does "Caring for child with CP is a way of life" mean<br>• How children with CP change over time<br>• How we can do everyday things in a way that is helpful to children with CP<br>• Helpful ways to hold and move children with CP<br>• Helpful ways to put children with CP in different positions
3	Getting my child's body ready to move and be active	• How to get a child's body ready to move and play<br>• How to position a child in a helpful way<br>• How to use these ideas in everyday activities
4	Eating and drinking: making mealtimes safe and comfortable for my child	• How to best position a child with CP for feeding<br>• Why it is important to feed a child with CP slowly<br>• How to prepare the child's lips and mouth for eating<br>• How to help a child with CP to eat better with a spoon<br>• How to help a child with CP to learn to chew<br>• How to help a child feed himself<br>• How to help a child with CP learn to drink from a cup<br>• How to help children who drool
5	Communication: my child and I understanding each other	• What we mean by communication<br>• The different ways we can communicate<br>• How to encourage a child with CP to communicate<br>• How to help a child with CP to understand better<br>• How to help a child with CP to talk better<br>• Helping children to use signs
6	Play: unlocking my child's potential	• What is play and why it is important for a child with CP<br>• How children usually play<br>• Why it is difficult for children with CP to play<br>• How we can make it easier for all children with CP to play<br>• What we can play with
7	CVI: Understanding where and what my child can see	• Understand vision difficulties<br>• What does CVI stand for and what does it mean<br>• How to know if a child has a CVI<br>• How to play with a child with a CVI<br>• How we use what the child sees best in everyday life

Table 1: Parent led workshops based on Malamulele Onward Carer-2-Carer Training Programme (each workshop runs for 2 – 2 ½ hours).

To implement the Akwenda CP programme, four caregivers (three mothers and one aunt) from Iganga were identified based on their availability, motivation, initiative, interest, and communication skills. They received four weeks training from a master trainer from Malamulele Onward, herself a parent of a child with CP.

Each of the seven trainings is approximately two hours long and will be led by two Iganga Parent Facilitators working as a team. To ensure quality and consistency of the workshops, the Parent Facilitators will follow the detailed Malamulele Onward Carer-2-Carer Training Programme manual which has been translated into Lusoga, the local language. For each workshop the Parent Facilitators have a workshop kit, which includes display materials. No therapist will be involved in these seven caregiver-led workshops.

Component 2: Therapist-led practical group sessions and workshops

Two weeks following each caregiver-led workshop, caregivers and children will attend a therapist-led practical group sessions that will have the same themes as the caregiver-led training workshop (Table 1). The therapists will be assisted by the Parent Facilitators during these practical sessions. Active parent participation is an essential feature of the group practical sessions which will be structured in such a way that the therapist will be able to give each child individual attention during the group activities.

In addition, the therapists involved in the intervention will run four group training workshops on: i) technical assistive devices; ii) goal directed training and setting realistic goals; iii) nutrition and iv) epilepsy (Table 2). In the previous study on this cohort of children with CP we noted that almost half (46%) of the children were severely malnourished, and 35% of the children suffered from seizures.[13] A manual for these sessions have been developed to ensure quality and consistency.

	Title of workshop	Outcomes
1	Healthy diet and healthy eating	• Know the seven major classes of nutrients<br>• Know how to prepare a meal from locally available nutrient dense foods<br>• Understand responsive feeding practices (e.g. frequency of feeding; creating a safe and clean environment for feeding)
2	Understanding epilepsy	• How to recognise a seizure<br>• Understand misconceptions related to epilepsy and how to deal with stigma<br>• Know when and where to seek care for a child with seizures<br>• Understand the role of traditional healers and traditional treatments<br>• Understand anti-epileptic medicine – how to administer; side effects and the importance of adherence

Table 2: Therapist-led training workshops on nutrition and seizures (each workshop is one hour long)

The two therapists running these practical sessions are experienced in working with children with CP in rural areas of Uganda, and have received 12 days of training on the practical management of children with CP. This training included essential concepts and components of the intervention programme viz., functional activities; the child's active participation and the value of self-generated movements; goal setting; working in groups; working in partnership with parents; technical assistive devices; nutrition and epilepsy. A therapist manual detailing the structure and content of each practical session has been drawn up for the intervention.

Component 3: Provision of technical assistive devices (TAD)

Provision of technical assistive devices (TAD) is an important part of evidence based best practice as they promote greater independence and enables children to perform tasks they were formerly unable to accomplish within the domains of mobility, self-care, eating and communication.[14] In this study, the aim of provision of TAD will be to simplify caregiving and increase the child's activity and participation.

The therapists will together with the caregivers and children assess the needs of TAD during the baseline assessments. Children and youth with lower limb contractures who would benefit from the provision of a standing frame or walking device will be referred to the local hospital for serial casting if it is thought that serial casting will provide sufficient soft tissue length to enable the child or youth to stand in alignment and with their feet in an acceptable position.

Where possible, TAD will be locally manufactured with affordable local materials, and the caregiver will be involved in cost sharing to ensure affordability and sustainability. Postural mobility devices, e.g., wheelchairs or buggies are costly and difficult to make locally, and will be procured from the Ministry of Health's accredited assistive devices production unit and/or donor agencies.

Children at GMFCS III-IV will be provided with standing frames, customised posture support chairs, walking assistive device and parallel bars f all of which will be made for use in the home environment by local artisans from either wood or using appropriate paper technology. Simple communication books or boards will be provided for children with communication difficulties. Children and youth will be issued with re-usable nappies or sanitary pads if needed. Other devices will be procured or created by the treating therapist in collaboration with the primary caregiver as the need arises, such as the construction of an accessible toilet seat, cut out feeding cups, shoes, splints, and appropriate toys.

The TAD will be issued during the 3rd month of the intervention following a therapist-led training workshop explaining the use, maintenance and care of assistive devices. The therapist together with the community social worker will deliver the TAD to the child's home where they will also ensure correct fitting of the device. Usage of the TAD will be evaluated during a home visit in the 8th month.

Component 4: Goal directed training

Goal directed training is a child-active, motor learning approach, and includes daily practice directly targeting the achievement of a goal.[15] The child and family, supported by health workers, identify realistic goals and create action plans for how to achieve them. Several studies have demonstrated the success of goal directed programs in HIC,[16-19] while there are no reports from LMIC. The goal-setting procedure in the Akwenda CP programme will start during the fourth month when the therapist has studied the assessment results of the child and become acquainted with the child and caregiver and the caregiver has attended the first two caregiver-led training workshops, meaning that she/he has a better understanding of the child's condition and potential. The therapist will lead two consecutive group sessions to explain the concept of goal setting to parents, during which the parents will assist one another in identifying goal-areas for their child. Following the group sessions, the therapists will visit each child in their home setting where the child and caregiver supported by the therapist will set three goals based on the goal-areas identified by the parents. An individualised activity plan will thereafter be made for each child, with the aim of achieving the goals within five months through daily training and practice in the home environment. Assessments performed before the intervention begins, including the Gross Motor Function Measurement (GMFM),[20] the Ugandan version of the Pediatric Evaluation of Disability Inventory (PEDI-UG), [21 22] and Picture my Participation measure,[23] will be used to inform the goal-setting procedure by identifying realistic goals based on the child's abilities and potential. These assessments explore different dimensions within the ICF framework, i.e., functional limitations, ability to perform activities and participation.

Each of the three goals will be scaled by the therapist as goal attainment scale (GAS) goals. For children issued with TAD, a fourth GAS goal will relate to the use of these TAD. Three months later, all GAS goals and the individualised activity plans for each child will be reviewed during the monthly group session or at a home visit; and re-evaluated in the 11th month.

Component 5: Communication and Advocacy for behavioural and social change (C&A)

This component is based on Communication for Development (C4D), a systematic, planned and evidence-based strategy to promote positive social and behavioural changes.[24] It addresses the stigma, discrimination and exclusion of children with disability and forms an important part in making the Akwenda cerebral palsy programme accessible and acceptable. Target groups include community leaders, healthcare professionals, school educators, the general population, families, and the caregivers themselves.

Caregivers of the children with CP in the intervention group will participate in three C&A meetings to be conducted in months one, two, and six of the intervention. Each meeting will last 90 minutes and be led by the community social worker. It will take place on the same day as one of the parent-led workshops. Topics for these meetings are (i) rights of children with disabilities with a focus on human rights and abuses of children with CP in the community;

(ii) mapping available health, education and legal services in the community; and (iii) barriers to services and how to overcome those barriers. These discussions and the proposed solutions will then be discussed with the community leaders at a joint dialogue session the 7th month.

Forty community representatives, including political, administrative, cultural and religious leaders, educators, health workers, police, disability NGO/CBO representatives, district and sub-county technical personnel will be invited to participate in two C&A meetings led by the community social worker. The first session will focus on the rights of children with disabilities and the stigma they meet leading to discrimination and barriers limiting their participation. The second will be a joint session with the caregivers discussing how to make services inclusive and to develop a community action plan. The community social worker will follow up the implementation of the community action plan in months nine to eleven of the intervention through regular telephone feedback from participants and/or visits to the participating communities.

In preparation for the C & A component and to sensitize the community to disability issues, information on cerebral palsy will be delivered through a one-hour talk show on six community radio stations by the community social worker. Messages will include information relating to how the CURIE III study intends to assist children with cerebral palsy in Iganga-Mayuge. A telephone number of the community social worker will be shared with the listeners at the end of each radio talk show.

Time table and delivery of the integrated intervention

The time table for delivering the intervention through the five activities is shown in Figure 3. It will be delivered over a period of 11 months with caregivers attending two sessions per month, one of which will be together with the child. In addition, there will be three home visits and five C&A sessions. Two therapists, four parent facilitators and one community social worker will deliver the services required.

Discussion

The aim of the Akwenda CP intervention programme for children and youth with CP is to provide a comprehensive spectrum of interventions for the child, the caregiver and the community. The core of the intervention is a carer-to-carer training programme. Parent training programmes have previously been reported from Ghana and West Bengal, [7 8] however, that training was either delivered by health workers or community disability workers, only some of whom were mothers of children with disabilities.

In order to make this intervention holistic and comprehensive to impact the child, the caregiver and the wider community, the carer-to-carer training has been complemented with additional activities aiming to improve children's functional abilities, activities and participation as well as a community and advocacy programme aiming at social and behavioural changes in family and community. Good clinical practice should strive to address

the various ICF domains at the same time since improvements in one domain may not transfer directly to others.[25]

In line with WHO's goal of universal health coverage, we have tried to make the Akwenda CP programme physically accessible, affordable and acceptable. The venue for the intervention is a community setting within easy reach of the children and caregivers, making it physically accessible in a timely manner with minimal transportation costs. The intervention is designed to be affordable for caregivers living in a low resource setting where many are living at or below the poverty level, and with a scarcity of therapists and other rehabilitation professionals. Therapists are essential but their skills and time need to be optimised and leveraged, and the use of caregivers as in this programme, is one way to achieve this. TAD are largely locally manufactured.

Stigma and discrimination increases the risk that children with CP are neglected by primary health care and education services. The communication and advocacy component aims at social and behavioural change in the local community but particularly targeting health workers, teachers and community leaders. Empowerment of children and parents will increase their confidence and emphasize their rights to receive basic health and rehabilitation services. Working with caregivers and children in group settings, as opposed to working on an individual one to one setting, will also unify parents in similar situations and strengthen their confidence.

Currently, there are few evidence-based interventions that have been evaluated in LMICs. The activities of the Akwenda CP intervention programme is therefore based on best clinical practice derived from clinical studies in HIC, and adapted to the conditions in this rural part of Uganda in accordance with the WHO's goal of universal health coverage and universal access.[9] The multiple activities add to the complexity of the intervention as well as its evaluation. This study will provide information on the affordability and cost-benefit ratio of the provision of a comprehensive intervention in low-resource setting.

References:

1. Onward M. CARER-2-CARER TRAINING PROGRAMME 2020 [Available from: https://www.malamuleleonward.org/what-we-offer/carer-2-carer-programme.
2. Saloojee G, Bezuidenhout M. Parent-led services and community-based peer supporters for children and adults with disabilities: experiences from two programmes. In: Padarath A, Kathard H, Lorenzo T, et al., eds. South African Health Review 2020. Durban: Health Systems Trust 2020.
3. World Health Organization. International classification of functioning, disability and health: children and youth version: ICF-CY 2007 [Available from: https://apps.who.int/iris/handle/10665/43737.
4. King G, Williams L, Goldberg SH. Family-oriented services in pediatric rehabilitation: a scoping review and framework to promote parent and family wellness. *Child Care Hlth Dev* 2017;43(3):334-47. doi: 10.1111/cch.12435
5. Novak I, McIntyre S, Morgan C, et al. A systematic review of interventions for children with cerebral palsy: state of the evidence. *Dev Med Child Neurol* 2013;55(10):885-910. doi: 10.1111/dmcn.12246

6. Diaz Heijtz R, Forssberg H. Translational studies exploring neuroplasticity associated with motor skill learning and the regulatory role of the dopamine system. *Dev Med Child Neurol* 2015;57 Suppl 2:10-4. doi: 10.1111/dmcn.12692
7. Benfer KA, Novak I, Morgan C, et al. Community-based parent-delivered early detection and intervention programme for infants at high risk of cerebral palsy in a low-resource country (Learning through Everyday Activities with Parents (LEAP-CP): protocol for a randomised controlled trial. *BMJ Open* 2018;8(6):e021186. doi: 10.1136/bmjopen-2017-021186
8. Zuurmond M, Nyante G, Baltussen M, et al. A support programme for caregivers of children with disabilities in Ghana: Understanding the impact on the wellbeing of caregivers. *Child Care Health Dev* 2019;45(1):45-53. doi: 10.1111/cch.12618
9. Evans DB, Hsu J, Boerma T. Universal health coverage and universal access: World Health Organization; 2013 [Available from: http://dx.doi.org/10.2471/BLT.13.125450 [PDF].
10. Kakooza-Mwesige A, Andrews C, Peterson S, et al. Prevalence of cerebral palsy in Uganda: a population-based study. *Lancet Glob Health* 2017;5(12):e1275-e82. doi: 10.1016/S2214-109X(17)30374-1
11. Andrews C, Kakooza-Mwesige A, Almeida R, et al. Impairments, functional limitations, and access to services and education for children with cerebral palsy in Uganda: a population-based study. *Dev Med Child Neurol* 2019 doi: 10.1111/dmcn.14401
12. Kakooza-Mwesige A, Tumwine JK, Eliasson AC, et al. Malnutrition is common in Ugandan children with cerebral palsy, particularly those over the age of five and those who had neonatal complications. *Acta Paediatr* 2015;104(12):1259-68. doi: 10.1111/apa.13089
13. Andrews C, Kakooza-Mwesige A, Almeida R, et al. Impairments, functional limitations, and access to services and education for children with cerebral palsy in Uganda: a population-based study. *Dev Med Child Neurol* 2020;62(4):454-62. doi: 10.1111/dmcn.14401
14. WHO. Global Cooperation on Assistive Technology (GATE) 2020 [Available from: https://www.who.int/disabilities/technology/gate/en/.
15. Ketelaar M, Vermeer A, Hart H, et al. Effects of a functional therapy program on motor abilities of children with cerebral palsy. *Phys Ther* 2001;81(9):1534-45. doi: 10.1093/ptj/81.9.1534
16. Ahl LE, Johansson E, Granat T, et al. Functional therapy for children with cerebral palsy: an ecological approach. *Dev Med Child Neurol* 2005;47(9):613-9.
17. Bower E, McLellan DL, Arney J, et al. A randomised controlled trial of different intensities of physiotherapy and different goal-setting procedures in 44 children with cerebral palsy. *Dev Med Child Neurol* 1996;38(3):226-37. doi: 10.1111/j.1469-8749.1996.tb15084.x
18. Lowing K, Bexelius A, Brogren Carlberg E. Activity focused and goal directed therapy for children with cerebral palsy--do goals make a difference? *Disabil Rehabil* 2009;31(22):1808-16. doi: 10.1080/09638280902822278
19. Lowing K, Bexelius A, Carlberg EB. Goal-directed functional therapy: a longitudinal study on gross motor function in children with cerebral palsy. *Disabil Rehabil* 2010;32(11):908-16. doi: 10.3109/09638280903353422
20. Russell DJ, Avery LM, Rosenbaum PL, et al. Improved scaling of the gross motor function measure for children with cerebral palsy: evidence of reliability and validity. *Phys Ther* 2000;80(9):873-85.
21. Kakooza-Mwesige A, Tumwine JK, Forssberg H, et al. The Uganda version of the Pediatric Evaluation of Disability Inventory (PEDI). Part I: Cross-cultural adaptation.

Child Care Health Dev 2018;44(4):552-61. doi: 10.1111/cch.12563 [published Online First: 2018/03/13]

22. Amer A, Kakooza-Mwesige A, Jarl G, et al. The Ugandan version of the Pediatric Evaluation of Disability Inventory (PEDI-UG). Part II: Psychometric properties. *Child Care Health Dev* 2018;44(4):562-71. doi: 10.1111/cch.12562
23. Arvidsson P, Dada S, Granlund M, et al. Content validity and usefulness of Picture My Participation for measuring participation in children with and without intellectual disability in South Africa and Sweden. *Scand J Occup Ther* 2019:1-13. doi: 10.1080/11038128.2019.1645878
24. UNICEF. UNICEF 2017 Report on Communication for Development (C4D) 2017 [Available from: https://www.unicef.org/media/47781/file/UNICEF_2017_Report_on_Communication_for_Development_C4D.pdf.
25. Novak I, Morgan C, Fahey M, et al. State of the Evidence Traffic Lights 2019: Systematic Review of Interventions for Preventing and Treating Children with Cerebral Palsy. *Curr Neurol Neurosci Rep* 2020;20(2):3. doi: 10.1007/s11910-020-1022-z
