# Supplementary material for: Akwenda intervention programme for children and youth with cerebral palsy in a low-resource setting in sub-Saharan Africa: protocol for a quasi-randomised controlled study
Source: BMJ Open. 2021 Mar 8;11(3):e047634. doi: 10.1136/bmjopen-2020-047634 (PMC7942240; doi:10.1136/bmjopen-2020-047634)
Supplement: Author's manuscript [file bmjopen-2020-047634.draft_revisions.pdf]

BMJ Open is committed to open peer review. As part of this commitment we make the peer review history of every article we publish publicly available.

When an article is published we post the peer reviewers' comments and the authors' responses online. We also post the versions of the paper that were used during peer review. These are the versions that the peer review comments apply to.

The versions of the paper that follow are the versions that were submitted during the peer review process. They are not the versions of record or the final published versions. They should not be cited or distributed as the published version of this manuscript.

BMJ Open is an open access journal and the full, final, typeset and author-corrected version of record of the manuscript is available on our site with no access controls, subscription charges or pay-per-view fees (http://bmjopen.bmj.com).

If you have any questions on BMJ Open's open peer review process please email info.bmjopen@bmj.com

BMJ Open

Community Based Intervention Programme for Children with Cerebral Palsy in a Low-Resource Setting in Sub-Saharan Africa: protocol for a quasi-randomized controlled study

Journal:	*BMJ Open*
Manuscript ID	bmjopen-2020-047634
Article Type:	Protocol
Date Submitted by the Author:	03-Dec-2020
Complete List of Authors:	Saloojee, Gillian; Independent researcher<br>Ekwan, Francis; Mulago National Referral Hospital, Department of Occupational Therapy,<br>Andrews, Carin; Karolinska Institutet, Department of Women's and Children's Health<br>Damiano, Diane; The National Institutes of Health Clinical Cente, Rehabilitation Medicine Department<br>Kakooza, Angelina; Mulago Hospital/Makerere University, Department of Paediatrics and Child Health<br>Forssberg, Hans; Karolinska Institutet, Women's and Children's Health
Keywords:	Developmental neurology & neurodisability < PAEDIATRICS, REHABILITATION MEDICINE, Community child health < PAEDIATRICS

SCHOLARONE™
Manuscripts

I, the Submitting Author has the right to grant and does grant on behalf of all authors of the Work (as defined in the below author licence), an exclusive licence and/or a non-exclusive licence for contributions from authors who are: i) UK Crown employees; ii) where BMJ has agreed a CC-BY licence shall apply, and/or iii) in accordance with the terms applicable for US Federal Government officers or employees acting as part of their official duties; on a worldwide, perpetual, irrevocable, royalty-free basis to BMJ Publishing Group Ltd ("BMJ") its licensees and where the relevant Journal is co-owned by BMJ to the co-owners of the Journal, to publish the Work in this journal and any other BMJ products and to exploit all rights, as set out in our licence.

The Submitting Author accepts and understands that any supply made under these terms is made by BMJ to the Submitting Author unless you are acting as an employee on behalf of your employer or a postgraduate student of an affiliated institution which is paying any applicable article publishing charge ("APC") for Open Access articles. Where the Submitting Author wishes to make the Work available on an Open Access basis (and intends to pay the relevant APC), the terms of reuse of such Open Access shall be governed by a Creative Commons licence – details of these licences and which Creative Commons licence will apply to this Work are set out in our licence referred to above.

Other than as permitted in any relevant BMJ Author's Self Archiving Policies, I confirm this Work has not been accepted for publication elsewhere, is not being considered for publication elsewhere and does not duplicate material already published. I confirm all authors consent to publication of this Work and authorise the granting of this licence.

For peer review only

Title:

Akwenda intervention programme for children and youth with cerebral palsy in a low-resource setting in sub-Saharan Africa: protocol for a quasi-randomised controlled study.

Authors:

Gillian Saloojee, Independent researcher, Johannesburg, South Africa
E-mail: gillian.saloojee@gmail.com

Francis Ekwan, Principal Occupational Therapist, Department of Occupational Therapy, Mulago National Referral Hospital, Kampala, Uganda
E-mail: ekwanfr@yahoo.com

Carin Andrews, Neuropaediatrics, Department of Women's and Children's Health, Karolinska Institutet, Stockholm, Sweden
E-mail: carin.andrews@ki.se

Diane Damiano, National Institutes of Health, Bethesda, MD, USA.
E-mail: damianod@cc.nih.gov

Angelina Kakooza-Mwesige, Department of Paediatrics and Child Health, Makerere University College of Health Sciences, Kampala, Uganda
E-mail: angelina_kakooza@yahoo.co.uk

Hans Forssberg, Neuropaediatrics, Department of Women's and Children's Health, Karolinska Institutet, Stockholm, Sweden
E-mail: hans.forssberg@ki.se

Corresponding author:

Hans Forssberg, Neuropaediatrics, Department of Women's and Children's Health, Astrid Lindgren Children's Hospital, Karolinska Institutet, 1776 Stockholm, Sweden
E-mail: hans.forssberg@ki.se
Phone: +46 708 730830

Keywords:
Cerebral palsy, community based intervention, family-oriented services, communication and advocacy, low and middle-income country

Word count:
Abstract: 300 words
Main article: 3 983 words

For peer review only

Abstract

Introduction

Cerebral palsy (CP) is the most common childhood-onset motor disorder accompanied by associated impairments placing a heavy burden on families and health systems. Most children with CP live in low- and middle-income countries (LMIC) with little access to rehabilitation services. This study will evaluate the Akwenda CP programme, a multi-dimensional intervention designed for low resource settings and aiming at improving: i) participation, motor function and daily activities for children with CP; ii) quality of life, stress and knowledge for caregivers; and iii) knowledge and attitudes towards children with CP in the communities.

Methods

This quasi-randomized controlled, clinical study will recruit children and youth with CP aged 2-23 in a rural area of Uganda. Children will be allocated to one of two groups with at least 44 children in each group. Groups will be matched for age, sex and motor impairment. The intervention arm will get a comprehensive, multi-dimensional programme over a period of 11 months comprising i) caregiver-led training workshops, ii) therapist-led practical group sessions, iii) provision of technical assistive devices, iv) goal based training and v) community communication and advocacy. The other group will receive usual care. The outcome of the intervention will be assessed before and after the intervention and will be measured at three levels, (i) child, ii) caregiver, and iii) community. Standard analysis methods for randomised controlled trial will be used to compare groups. Retention of effects will be examined at 12-month follow-up.

Ethics and dissemination

The study has been approved by the Uganda National Council for Science and Technology (UNCST) (SS 5173) and registered in accordance with WHO and ICMJE standards at the Pan African Clinical Trials Registry (PACTR202011738099314). Written informed consent will be obtained from caregivers. Results will be disseminated among participants and stakeholders through public engagement events, scientific reports and conference presentations.

For peer review only - http://bmjopen.bmj.com/site/about/guidelines.xhtml

Strengths and limitations of this study

- This is the first registered randomized controlled clinical trial of a comprehensive, multi-dimensional intervention programme for children and youth with CP in a low and middle-income country.
- The core of the programme, based on principles derived from the International Classification of Functioning, Disability and Health (ICF), evidence based interventions and universal health coverage, will be parent-led training workshops which accounts for the scarcity of rehabilitation therapists and specialists.
- The study will address several ICF levels and include a battery of objective, validated and adapted outcome measures that will measure a broad spectrum of intervention outcomes at the level of the child, the caregiver and the community.
- The small sample size will make it difficult to identify small effects and it will not be possible to identify which of the components is most effective.

For peer review only

For peer review only - http://bmjopen.bmj.com/site/about/guidelines.xhtml

INTRODUCTION

Cerebral palsy (CP) is a life-long motor disorder due to non-progressive disturbances of the developing foetal or infant brain. [1] In addition to gross and fine motor impairments, children with CP often have associated impairments in hearing, vision, cognition and communication, as well as behavioural problems and seizures. Feeding difficulties result in a high prevalence of malnutrition. [2] Apart from access to main stream health care, social services and education, children with CP require rehabilitation services for their special needs. These needs are not met in many low- and middle-income countries (LMICs) where children with developmental disabilities often are neglected by primary health care and education services and face discrimination and exclusion.[3 4] Discrimination often stems from a lack of knowledge, prejudice and cultural norms, which in turn lead to stigma and entrenched social exclusion.[5-7] LMICs are characterised by a paucity of health professionals specialized in rehabilitation including physio-, occupational- and speech and language therapists,[8, 9 10] as well as a lack of assistive devices.[11] Professional consensus reports from Africa illustrate an immense lack of services for children with neurodevelopmental disorders with limited health worker and caregiver awareness, poor community attitudes and practices, lack of health worker training with unclear treatment or referral guidelines and lack of diagnostic facilities or feasible health interventions.[12-14]

The scarcity of rehabilitation services has resulted in families being left alone to care for the child with CP, neither knowing the cause and the life-long prognosis, nor given any advice how to best care for the child. This situation creates a high risk for stress, isolation, depression and reduced quality of life for the caregivers. [15-18] In the absence of health professionals, training of family members has become a strategy to address the workforce gap, for example the *Care Skills Training* programs for children with developmental delays and disabilities.[19] A recent initiative for CP in LMIC is a caregiver training package (*Getting to know cerebral palsy; GTKCP),*[20] which aims to empower caregivers and improve care and support for children, recently studied in Ghana.[21 22] A comprehensive parent-delivered intervention program for early intervention for infants at high risk to develop CP, (*Learning through Everyday Activities with Parents; LEAP_CP)* is currently being evaluated in a randomized controlled trial in Bangladesh.[23] In addition to parent education, LEAP_CP comprises goal directed motor and cognitive skill training and strategies to enrich the home environment. Another early intervention parent-delivered programme, the “ABAaNA EIP” is currently being trialled in Uganda for infants under 12 months.[24] There are thus several emerging intervention programmes for young children with CP in low resource settings based on evidence derived from high-income countries (HIC), although as yet, no evidence based programme has been tested in LMICs.

The neglect and lack of services and support for children with CP in LMIC is underscored by limited information about the epidemiology of CP in these countries. In 2015, our research team published the first population-based study on children with CP in sub-Saharan Africa, reporting a 50% higher prevalence than HIC (a crude prevalence of 3·1/1000, [25] vs. 1·8–2·3 in HIC [26-29]). The finding of higher prevalence of CP has been confirmed in a recent community-based key informant study from Bangladesh [30 31] and is consistent with some previous

For peer review only - http://bmjopen.bmj.com/site/about/guidelines.xhtml

estimates from different LMIC populations. [32-35] Many children in our study had severe motor impairments often in combination with intellectual disability, impairments in communication, and seizures. None had received regular rehabilitation services. Only 8% of those unable to walk had wheelchairs, and none had assistive devices for walking, hearing, seeing or communication. Only a third of the children attended school. Notably, the Ugandan cohort of children with CP exhibited significantly lower developmental trajectories of mobility and self-care skills when compared to a similar CP cohort of Swedish children. We concluded that the needs for children with CP and their families in rural Uganda were not met, illustrated by the scarcity of health services, assistive devices, and low school attendance, and that the absence of interventions and support contributed to the poorer development of mobility and self-care skills.[4]

To address this unsettling situation, we have developed an intervention programme for children with CP living in rural, low-resource settings, which we plan to implement and evaluate in this cohort of children with CP in rural Uganda. This Akwenda CP programme was developed by an international team of experts on CP and rehabilitation, an academic team from Uganda, a team from Malamulele Onward in South Africa and a local team of therapists and social workers living in the region in a three step process described in *Supplement 1*. The first step involved identifying key principles based on current concepts of disability, rehabilitation and interventions for children with CP. In the second step the team set up three general programme goals based on the principles and the needs of children with CP and their families, which had been identified in previous studies.[2 25 36] In the third step, the team developed the Akwenda CP programme that comprises five components, based on the principles and aimed at achieving the goals formulated in the previous steps (See *Supplement 1, Supplemental Figure 1, and Supplemental Tables 1 & 2*).

Aims and hypotheses

This protocol describes the method and implementation of the Akwenda CP programme study, which aims to evaluate the programmes feasibility and impact on children, caregivers and society. The specific aims are to:

1. Determine the efficacy of the intervention on the motor function, daily activities and participation of children with CP.
   Hypothesis 1: Children with CP receiving the intervention will improve their motor function, enhance their activity and increase their level of participation compared with children receiving usual care.

2. Describe the impact of the intervention on the behaviour and mental health of caregivers of children with CP.
   Hypothesis 2: Caregivers of children with CP receiving intervention will have a better quality of life, reduced levels of anxiety and stress, and increased levels of knowledge and practical caregiver competence compared with caregivers receiving usual care.

For peer review only - http://bmjopen.bmj.com/site/about/guidelines.xhtml

3. Determine the impact of the intervention on the community awareness of childhood disability and attitudes towards children with disability and their families.
   Hypothesis 3: Stakeholders, teachers, health professionals and the lay community will have increased their knowledge about childhood disability and the barriers limiting their participation in society. Their perception of disability will become more positive.

Methods

Study design

The project is designed as a quasi-randomized controlled, clinical study conducted according to SPIRIT guidelines (see Figure 1). Children with confirmed diagnoses of CP will be assigned to either a study group or control group, based on geographic regions. The study group will receive the Akwenda CP programme during the first year, whilst the control group will be on a waitlist and receive usual care. The control group will receive the intervention after 12 months. Children and care givers of the first intervention group will be encouraged to continue with the parent-led workshops and to implement new caregiving skills and behaviours during the second year. Both groups will be assessed prior to the start of the intervention, after one year and after two years.

FIGURE 1 HERE

Study site

The study will be performed at the Iganga/Mayuge Health Demographic Surveillance Site which was set up in collaboration between Makerere University and Karolinska Institute. It comprises 65 villages including 15,964 households, and >85 000 individuals of whom approximately 50% are under 18 years. The populations are predominantly rural subsistence farmers living under the poverty level, but 20% of the population resides in the semi-urban Iganga town. The HDSS includes one general hospital (Iganga Hospital), 27 health centres and 132 private health providers.

Participants and recruitment

Participants will be from a population-based cohort of children with CP identified from previous screening carried out at IM-HDSS in 2015.[25] In addition, children with CP aged 2-6 years living in the same area will be recruited by the community development officer. Hence, the study population will be between 2 and 23 years at the onset of the intervention study.

For peer review only

Randomisation and blinding

Since the community and advocacy intervention will target the community, all children from a particular village need to be in the same group. Children will thus be allocated to groups depending on the village where they reside. Neighbouring villages will be clustered together to reduce contamination between groups resulting in two geographically defined groups, which will be matched regarding age, sex and motor function. The decision of which group will receive the intervention first will be decided by a coin flip. A therapist team and social worker will administer the assessments and surveys before the study to capture background and baseline data. A group of independent therapists, not involved in the intervention, will administer the outcome measures after the interventions. These examiners will be masked to group allocation.

Intervention arm

The Akwenda CP programme comprises five elements (See supplement and Supplemental Tables for detailed description):

Caregiver-led training workshops: The core is a series of seven workshops for caregivers of children with CP. This workshop series is based on the Malamulele Onward Carer-2-Carer Training Programme (MOC2CTP).[37 38] Four caregivers of children with CP from Iganga have been identified and trained to lead the workshops following the detailed MOC2CTP-manual. (See Supplemental table 1).

Therapist-led practical group sessions and workshops: Trained paediatric therapists will run monthly practical sessions for groups of caregivers, based on the material covered in the caregiver-led workshops. The emphasis of the practical sessions is on active caregiver participation and includes training caregivers to facilitate their children's active participation and engagement. The sessions will be structured such that the therapist will be able to give each child individual attention during group activities. The parent facilitators will assist the therapists during these practical sessions. The therapists will in addition run four workshops including i) TAD; ii) goal based training; iii) nutrition and iv) epilepsy (See supplemental table 2). To ensure consistency and quality, a therapist manual was developed for the intervention.

Provision of technical assistive devices (TAD): TAD are an important part of evidence based rehabilitation, promoting greater independence and enabling children to perform tasks they were formerly unable to accomplish within mobility, self-care, eating and communication domains.[39] Each child's need for TAD will be assessed by therapists at baseline and the children and caregivers will be involved in deciding what TAD they need. Examples are wheelchairs, standing frames, posture support chairs, walking devices, parallel bars, communication boards and cut out feeding cups. When possible, TAD will be locally

For peer review only - http://bmjopen.bmj.com/site/about/guidelines.xhtml

manufactured with affordable local materials, and caregivers will be involved in cost sharing to ensure affordability and sustainability.

Goal setting: Goal directed therapy is a child-active, motor learning approach. [40] Several studies have demonstrated successful goal directed programs in HIC,[41-44] while there are no reports from LMIC. The child and caregiver, supported by a therapist, will identify three realistic goals and create action plans for how to achieve them through daily training and practice in the home environment. The goal-setting procedure will start during the fourth month. By this time the therapist has become acquainted with the child, and the caregiver has attended the caregiver-led workshops and will have a more realistic understanding of the child's potential. Children issued TAD will have a fourth goal relating to their use.

Communication and advocacy for behavioural and social change (C&A): The C&A component will address stigma, discrimination and exclusion of children with disabilities, and is based on Communication for Development (C4D), a systematic and evidence-based strategy to promote positive social and behavioural changes.[45] The broad themes for C&A are human rights of children with disabilities and barriers to services and how to overcome them. C&A will be run by a community social worker using community meetings and radio talk shows. Community meetings will involve political and religious leaders, teachers and health workers. Caregivers and community stakeholders will participate in a joint meeting to develop a community action plan for how to make services more inclusive.

Table 1: Time table of the intervention

Standard care arm

Standard or usual care refers to that available for children with CP in the study area. Currently, children have access to primary health care, the local hospital, and a newly opened NGO providing some rehabilitation services.[36] Caregivers in the usual care group will be asked to keep a monthly care diary during the waiting period.

Sample size

From the initial cohort of 97 children with CP, 82 children had survived. In order to include younger children, we have identified 20 additional children with CP living in the area. From these two samples, 88 children will be recruited to form two comparable groups each with 44 children. To explore whether this sample size was sufficient for detecting a true effect of the intervention, we did power calculations to determine what mean difference in PEDs-QL scores was needed between arms. We used a PEDs-QL mean of 12.5 and standard deviation of 18.6, from a previous study in Ghana on caregivers of children with CP.[22] In our population with 44 per group allowing for a 10% non-participation at follow up, a mean difference of at least 12.5 points between groups is needed for 80% power and a type I error of 0.05. Considering that the mean improvement after a parent training intervention in Ghana in PEDS-QL was +31.3 (95%CI 25.6-37.0) this seems plausible.[22]

For peer review only - http://bmjopen.bmj.com/site/about/guidelines.xhtml

Measures

To evaluate the multi-dimensional study aims, a series of outcome measures will be used before (baseline) and 1 and 2 years after the intervention at the following levels: i) child, ii) caregiver, and iii) community

Background characteristics

Background data will be collected on all children before allocation to groups. This includes sociodemographic information and medical history; Gross Motor Function Classification System (GMFCS); Manual Ability Classification System (MACS); Communication Function Classification System (CFCS), clinical and nutritional examinations including anthropometric data; information on school attendance, care seeking, and need for and current usage of TAD.

Child related outcome measures

Gross Motor Function Measure (GMFM66): The GMFM66 will be used as the primary outcome. It is a clinical tool designed to evaluate change in gross motor function in children with CP.[46]

Uganda version of Pediatric Evaluation of Disability Inventory (PEDI-UG): This a parent-reported measure of the child's independence in self-care, mobility and social function. The PEDI was developed in North America and has recently been culturally adapted and translated to the Ugandan context.[47 48]

Picture my Participation (PMP): PMP is an instrument designed to assess the participation of children, particularly in LMIC. [49] It measures attendance and involvement in 20 activities.

Goal attainment scale (GAS): GAS is an objective method of quantifying goal attainment that has been applied to children with CP. [40] Goals are scored on a 5-point, Likert-type scale from no change at all to attainment of goal at much more than the expected level. This measure will only be used in the intervention group.

Evaluation of TAD: A goal for the use of the assistive devices will be set for each child in the intervention group, and the GAS scale will be used to evaluate the use of assistive devices. User-satisfaction will be evaluated at the end of the intervention using a questionnaire based on the Quebec User Evaluation of Satisfaction with assistive Technology.[50]

Caregiver related outcome measures

A Knowledge Questionnaire evaluating caregiver's learning outcomes from the caregiver-led workshops will be administered before and after the workshop. This questionnaire comprises 26 statements, with three possible responses, viz., agree, disagree or not sure. It is based on the Knowledge Questionnaire used in the Malamulele Onward C2CTP and was modified during the piloting phase.

For peer review only - http://bmjopen.bmj.com/site/about/guidelines.xhtml

Caregivers skill and behaviours: Interaction, skills and behaviours of caregivers performing two everyday activities with their child: i.e., dressing and undressing; and feeding in the child's home will be captured in two videos, each four minutes long. Five observable behaviours will be scored using a four point Likert scale specifically developed by the research team for this study, with 0 being "none of the time" and 3 being "90% or more of the time."

Caregivers quality of life: Caregiver quality of life will be assessed using the 36 item Pediatric Quality of Life Inventory PedsQL Family Impact Module.[51] This was successfully used in a similar study in Ghana, [21] and has been translated into Luganda.

Caregivers stress: Caregiver stress will be assessed using the Zimbabwe Caregiver Challenges Scale.[52] This tool was recently developed for caregivers of children with lifelong disabilities living in low-resourced African settings.

Community related outcome measures

Communication and Advocacy Survey (CAS) Questionnaire: This tool is based on the CBM (Christian Blind Mission) Inclusive Development Tool Kit [53] and comprises 44 items measuring the knowledge, attitudes, and practices of community members. The tool, available in both English and Lusoga, has been developed by the research team and pre-tested on community members in Uganda.

Data collection, management and analysis

Organization and management

The Akwenda-CP programme is a study within the CURIE research consortium, a collaboration between the Makerere University College of Health Sciences, Kampala, Uganda and Karolinska Institute, Stockholm, Sweden and clinical researchers from South Africa and USA. The project will be guided by a steering group ensuring the conduct and coordination of the study. There will be a local Site Steering Committee consisting of the PI (AKM), an administrative project manager and a PhD student. The project manager will be coordinating the study on site and report to the PI.

Data collection and monitoring

Two therapists not involved in the intervention will administer the baseline and outcome assessments. They have pre-knowledge of the project from participating in the development of the intervention, and will be trained over a period of four weeks in the assessments. The PhD- student, also a therapist, will assist in the assessments and be responsible for data collection, storage and analysis. A data manager will supervise the overall organization, storage and security of the data and ensure information is recorded accurately.

For peer review only - http://bmjopen.bmj.com/site/about/guidelines.xhtml

Data management and analysis

Data will be collected in Open Data Kit using tablets, and electronic data will be managed through a secure database. Data will be analysed using SPSS. Standard analysis methods for a randomised controlled trial, including general linear mixed models, will be used to make comparisons between the intervention group and the wait list group on an intention to treat basis. Effect estimate will be presented as a mean difference and 95% CI. Secondary outcome analyses will use similar methods to compare all other outcomes between groups immediately post-intervention and again one year later. The GAS goal attainment scores for the intervention group will be analysed with paired t-tests on a within-group basis. A senior statistician will guide and assist with the analysis.

Patient and public involvement statement

The Akwenda CP programme and the study design have been developed in collaboration with caregivers, community leaders and local health professionals. Everyone was invited to a dissemination day in January 2019 where the results from the first screening study from 2015 were reported. At the same time, plans for a four-year follow-up study and this intervention study were presented and discussed. Local therapists and social workers from the region, together with four caregivers, have thereafter participated in the development and adaptation of the intervention programme (See supplement 1), and in designing and planning the study. The parent-led workshop series, a core part of the intervention programme, was developed and refined by a team of therapists and the parents of children with CP in South Africa.

Ethics and dissemination

Ethics: The research project has been reviewed by the Higher Degrees Research and Ethics Committee, School of Public Health, College of Health Sciences, Makerere University (Ref: 727) and approved by the Uganda National Council for Science and Technology (UNCST) (SS 5173). Written informed consent for study participation will be obtained from the mother or legal guardian, and, if possible, assent will be required from children above eight years. The electronic data will be managed through a secure database. Participant's identities will not be revealed in any publications or reports emanating from this study. Video file names will be de-identified. A study protocol including all items of the WHO Trial Registration Data Set has been completed and the study has been registered in accordance with WHO and ICMJE standards at the Pan African Clinical Trials Registry (PACTR202011738099314). Any amendment of the study protocol requires approval from the Research and Ethics Committee.

Safety: As the study is not a drug trial, there is no data safety monitoring committee. A COVID-19 risk management protocol has been developed and approved by the Higher Degrees Research and Ethics Committee, School of Public Health, College of Health Sciences, Makerere University and will be strictly adhered to. Any adverse events associated

For peer review only

with the study will be screened weekly by the treating therapists, and reported to the PI, and referred to the Ethics committee if considered serious. Any decision to describe interim results or terminate the trial will be made by the investigators with oversight from the Ethics committee.

Dissemination: Locally within Iganga-Mayuge, a stakeholders' dissemination event will be organized. Families involved in the study along with the key players in the health, community, policing, media, and disability will be invited to participate.

At the national level, findings will be disseminated through the Ministry of Health, Non Communicable Diseases (NCD) platform, and collaboration with health regulatory councils and professional associations will result in professional development courses that utilize the study findings to be disseminated to nearly all the health practitioners and health policy planners/implementers working in Uganda. Findings will also be disseminated through print media that will enable non-health workers to access information regarding the study findings.

For the international community, study findings will be published through Open Access, peer-reviewed scientific journals and at international conferences. Several authors are in the International Alliance of Academies of Childhood Disability (IAACD), and these findings will be important when developing recommendations for good clinical practice and will be disseminated through lectures and webinars organized by the IAACD.

DISCUSSION

To our knowledge, this is the first intervention programme for children and youth with CP in a LMIC that provides a holistic approach with a broad spectrum of interventions aimed at children and their caregivers as well as the wider community. The intervention is designed to be affordable in a low resource setting where many families live at or below the poverty level, and with a scarcity of therapists and other rehabilitation professionals. The core of the intervention is a carer-to-carer training programme. Intervention programmes in Ghana and West Bengal have previously used parent training programmes, [21 23] however, that training was either delivered by health workers or community disability workers, only some of whom were mothers of children with disabilities.

Currently, few evidence-based interventions have been evaluated in LMICs. The activities of the Akwenda CP programme are based on best clinical practice derived from clinical studies in HIC, and adapted to the conditions in this rural part of Uganda in accordance with the WHO's goal of universal health coverage and universal access.[54] The multiple activities add to the complexity of the intervention as well as the evaluation. It will therefore not be possible to study the effects of individual components. Our aim is rather to study the impact of the entire intervention as well as the programme's feasibility, cost implications, effectiveness, accessibility and acceptability.

Most intervention studies focus on children under the age of 12 years.[21-24] Our study includes a much wider age range, up to the age of 23 years. The majority of children and youth have not had access to early intervention or to regular therapy, which is the situation in many

For peer review only - http://bmjopen.bmj.com/site/about/guidelines.xhtml

LMICs. By including a wider age range of participants, findings from this study will be relevant to all age groups, not just younger children. Information from this study will also shed light on the extent to which positive change is still possible in youth with CP who have received very little intervention during early life.

Author contributions:

GS was leading the development of the intervention programme based on her previous experience at Malamulele Onward. She was also involved in designing the study protocol, drafted the manuscript and approved the final manuscript as submitted.

FE was involved in leading the intervention programme development, in particular the C&A component, and taking part in the design of the study protocol and drafted the manuscript and approved the final manuscript as submitted

CA has been involved in programme development and design of the study protocol and has revised drafts of the manuscript and approved the final manuscript as submitted.

DD has been involved in programme development and design of the study protocol and has revised drafts of the manuscript and approved the final manuscript as submitted.

AKM conceptualised the study and was involved in programme development and design of the study protocol and revised drafts of the manuscript and approved the final manuscript as submitted.

HF conceptualised the study, secured funding for the study, was involved in programme development and led the design of the study protocol and drafted the manuscript and approved the final manuscript and was responsible for submission.

Collaborators:

The development of the intervention was done in collaboration with a team of four local therapists from the region; Sauba Kamusiime OT, Catherine Ntanya OT, Edward Opio OT, and Eric Kintu PT, one social worker; Godfrey Wanjala from Iganga, four caregivers; Babra Esaete, Margret Nalweiso, Rose Naigaga, and Robinah Nakaziba who were trained to become Parent Facilitators by Lydia Ngwana, a master trainer from Malamulele Onward in Johannesburg, South Africa. The Parent Facilitators and master trainer were also involved in adapting the carer-to-carer programme to the local conditions in rural Uganda. Keron Ssebyla was involved in the project administration, coordination, and also supported the practical work in planning the intervention programme and study, and the training of the staff. Tom Basalanda was involved in coordinating logistical requirements during field activities including training. IM-HDSS supported with data management within their system at the Iganga field site.

Funding: We acknowledge the funding of the project by the Swedish Research Council (2017-05474) and by the Foundation Frimurare Barnhuset in Stockholm. The funders had no role in study design; collection, management, analysis, and interpretation of data; writing of the report; and the decision to submit the report for publication.

Competing interests: Non declared

Patient consent: Will be required for children and family participating in the study.

Data Availability Statement: This is a study protocol and as such no additional data is available. The data collected in the clinical trial used in the scientific reports will be uploaded at the Swedish National Data Service repository and shared without restrictions: SND-ID: 2020-181; Reserved DOI: 10.5878/97y5-5h14; https://snd.gu.se/en/catalogue/study/preview/4e2353f2-e277-42a1-8f0d-5281b3f22153

Open access: This is an open access article distributed in accordance with the Creative Commons Attribution 4.0 Unported (CC BY 4.0) license, which permits others to copy, redistribute, remix, transform and build upon this work for any purpose, provided the original work is properly cited, a link to the licence is given, and indication of whether changes were made. See: https:// creativecommons. org/ licenses/ by/ 4. 0/.

For peer review only

REFERENCES

1. Rosenbaum P, Paneth N, Leviton A, et al. A report: the definition and classification of cerebral palsy April 2006. *Dev Med Child Neurol Suppl* 2007;109:8-14. [published Online First: 2007/03/21]

2. Kakooza-Mwesige A, Tumwine JK, Eliasson AC, et al. Malnutrition is common in Ugandan children with cerebral palsy, particularly those over the age of five and those who had neonatal complications. *Acta Paediatr* 2015;104(12):1259-68. doi: 10.1111/apa.13089 [published Online First: 2015/06/20]

3. Kuper H, Monteath-van Dok A, Wing K, et al. The impact of disability on the lives of children; cross-sectional data including 8,900 children with disabilities and 898,834 children without disabilities across 30 countries. *PLoS One* 2014;9(9):e107300. doi: 10.1371/journal.pone.0107300

4. Andrews C, Kakooza-Mwesige A, Almeida R, et al. Impairments, functional limitations, and access to services and education for children with cerebral palsy in Uganda: a population-based study. *Dev Med Child Neurol* 2020;62(4):454-62. doi: 10.1111/dmcn.14401

5. Bunning K, Gona JK, Newton CR, et al. The perception of disability by community groups: Stories of local understanding, beliefs and challenges in a rural part of Kenya. *PLoS One* 2017;12(8):e0182214. doi: 10.1371/journal.pone.0182214

6. Kane JC, Elafros MA, Murray SM, et al. A scoping review of health-related stigma outcomes for high-burden diseases in low- and middle-income countries. *BMC Med* 2019;17(1):17. doi: 10.1186/s12916-019-1250-8

7. Mitter N, Ali A, Scior K. Stigma experienced by families of individuals with intellectual disabilities and autism: A systematic review. *Res Dev Disabil* 2019;89:10-21. doi: 10.1016/j.ridd.2019.03.001

8. Bright T, Wallace S, Kuper H. A Systematic Review of Access to Rehabilitation for People with Disabilities in Low- and Middle-Income Countries. *Int J Environ Res Public Health* 2018;15(10) doi: 10.3390/ijerph15102165

9. Gupta N, Castillo-Laborde C, Landry MD. Health-related rehabilitation services: assessing the global supply of and need for human resources. *BMC Health Serv Res* 2011;11:276. doi: 10.1186/1472-6963-11-276

10. World_Health_Organization. Rehabilitation 2030: A call for action 2017 [Available from: http://www.

who.int/disabilities/care/rehab-2030/en/

11. Matter R, Harniss M, Oderud T, et al. Assistive technology in resource-limited environments: a scoping review. *Disabil Rehabil Assist Technol* 2017;12(2):105-14. doi: 10.1080/17483107.2016.1188170

12. Donald KA, Kakooza AM, Wammanda RD, et al. Pediatric Cerebral Palsy in Africa: Where Are We? *J Child Neurol* 2015;30(8):963-71. doi: 10.1177/0883073814549245 [published Online First: 2014/10/10]

13. Wilmshurst JM, Badoe E, Wammanda RD, et al. Child neurology services in Africa. *J Child Neurol* 2011;26(12):1555-63. doi: 10.1177/0883073811420601

14. Scherzer AL, Chhagan M, Kauchali S, et al. Global perspective on early diagnosis and intervention for children with developmental delays and disabilities. *Dev Med Child Neurol* 2012;54(12):1079-84. doi: 10.1111/j.1469-8749.2012.04348.x

15. Gona JK, Mung'ala-Odera V, Newton CR, et al. Caring for children with disabilities in Kilifi, Kenya: what is the carer's experience? *Child Care Health Dev* 2011;37(2):175-83. doi: 10.1111/j.1365-2214.2010.01124.x [published Online First: 2010/07/22]

16. Hartley S, Ojwang P, Baguwemu A, et al. How do carers of disabled children cope? The Ugandan perspective. *Child Care Health Dev* 2005;31(2):167-80. doi: 10.1111/j.1365-2214.2004.00464.x

17. Mobarak R, Khan NZ, Munir S, et al. Predictors of stress in mothers of children with cerebral palsy in Bangladesh. *J Pediatr Psychol* 2000;25(6):427-33. doi: 10.1093/jpepsy/25.6.427

18. Dambi JM, Jelsma J, Mlambo T. Caring for a child with Cerebral Palsy: The experience of Zimbabwean mothers. *Afr J Disabil* 2015;4(1):168. doi: 10.4102/ajod.v4i1.168

19. WHO. Training parents to transform children's lives 2020 [Available from: https://www.who.int/mental_health/maternal-child/PST/en/.

20. GTKCP. Working with parent groups – a training resource for facilitators, parents, caregivers, and persons with cerebral palsy 2020 [Available from: https://www.lshtm.ac.uk/sites/default/files/2019-06/Getting-to-know-cerebral-palsy-english.pdf.

21. Zuurmond M, Nyante G, Baltussen M, et al. A support programme for caregivers of children with disabilities in Ghana: Understanding the impact on the wellbeing of caregivers. *Child Care Health Dev* 2019;45(1):45-53. doi: 10.1111/cch.12618

22. Zuurmond M, O'Banion D, Gladstone M, et al. Evaluating the impact of a community-based parent training programme for children with cerebral palsy in Ghana. *PLoS One* 2018;13(9):e0202096. doi: 10.1371/journal.pone.0202096

23. Benfer KA, Novak I, Morgan C, et al. Community-based parent-delivered early detection and intervention programme for infants at high risk of cerebral palsy in a low-resource country

For peer review only - http://bmjopen.bmj.com/site/about/guidelines.xhtml

(Learning through Everyday Activities with Parents (LEAP-CP): protocol for a randomised controlled trial. *BMJ Open* 2018;8(6):e021186. doi: 10.1136/bmjopen-2017-021186

24. Nampijja M, Webb E, Nanyunja C, et al. Randomised controlled pilot feasibility trial of an early intervention programme for young infants with neurodevelopmental impairment in Uganda: a study protocol. *BMJ Open* 2019;9(10):e032705. doi: 10.1136/bmjopen-2019-032705
25. Kakooza-Mwesige A, Andrews C, Peterson S, et al. Prevalence of cerebral palsy in Uganda: a population-based study. *The Lancet Global health* 2017;5(12):e1275-e82. doi: 10.1016/s2214-109x(17)30374-1 [published Online First: 2017/11/06]
26. Oskoui M, Coutinho F, Dykeman J, et al. An update on the prevalence of cerebral palsy: a systematic review and meta-analysis. *Developmental Medicine and Child Neurology* 2013;55(6):509-19. doi: 10.1111/dmcn.12080
27. Sellier E, Platt MJ, Andersen GL, et al. Decreasing prevalence in cerebral palsy: a multi-site European population-based study, 1980 to 2003. *Dev Med Child Neurol* 2016;58(1):85-92. doi: 10.1111/dmcn.12865
28. Van Naarden Braun K, Doernberg N, Schieve L, et al. Birth Prevalence of Cerebral Palsy: A Population-Based Study. *Pediatrics* 2016;137(1) doi: 10.1542/peds.2015-2872 [published Online First: 2015/12/15]
29. Smithers-Sheedy H, McIntyre S, Gibson C, et al. A special supplement: findings from the Australian Cerebral Palsy Register, birth years 1993 to 2006. *Dev Med Child Neurol* 2016;58 Suppl 2:5-10. doi: 10.1111/dmcn.13026
30. Khandaker G, Muhit M, Karim T, et al. Epidemiology of cerebral palsy in Bangladesh: a population-based surveillance study. *Dev Med Child Neurol* 2018 doi: 10.1111/dmcn.14013
31. Andrews C, Kakooza-Mwesige A, Eliasson AC, et al. Important report on cerebral palsy in Bangladesh: but different findings compared with other countries need further exploration. *Dev Med Child Neurol* 2019 doi: 10.1111/dmcn.14202
32. El-Tallawy HN, Farghaly WM, Shehata GA, et al. Cerebral palsy in Al-Quseir City, Egypt: prevalence, subtypes, and risk factors. *Neuropsychiatr Dis Treat* 2014;10:1267-72. doi: 10.2147/NDT.S59599
33. Chang MJ, Ma HI, Lu TH. Estimating the prevalence of cerebral palsy in Taiwan: A comparison of different case definitions. *Res Dev Disabil* 2014;36C:207-12. doi: 10.1016/j.ridd.2014.10.001
34. Banerjee TK, Hazra A, Biswas A, et al. Neurological disorders in children and adolescents. *Indian J Pediatr* 2009;76(2):139-46. doi: 10.1007/s12098-008-0226-z
35. Couper J. Prevalence of childhood disability in rural KwaZulu-Natal. *S Afr Med J* 2002;92(7):549-52.
36. Andrews C, Kakooza-Mwesige A, Almeida R, et al. Impairments, functional limitations, and access to services and education for children with cerebral palsy in Uganda: a population-based study. *Dev Med Child Neurol* 2019 doi: 10.1111/dmcn.14401 [published Online First: 2019/11/26]
37. Onward M. CARER-2-CARER TRAINING PROGRAMME 2020 [Available from: https://www.malamuleleonward.org/what-we-offer/carer-2-carer-programme.
38. Saloojee G, Bezuidenhout M. Parent-led services and community-based peer supporters for children and adults with disabilities: experiences from two programmes. In: Padarath A, Kathard H, Lorenzo T, et al., eds. South African Health Review 2020. Durban: Health Systems Trust 2020.

For peer review only - http://bmjopen.bmj.com/site/about/guidelines.xhtml

39. WHO. Global Cooperation on Assistive Technology (GATE) 2020 [Available from: https://www.who.int/disabilities/technology/gate/en/.

40. Ketelaar M, Vermeer A, Hart H, et al. Effects of a functional therapy program on motor abilities of children with cerebral palsy. *Phys Ther* 2001;81(9):1534-45. doi: 10.1093/ptj/81.9.1534

41. Ahl LE, Johansson E, Granat T, et al. Functional therapy for children with cerebral palsy: an ecological approach. *Dev Med Child Neurol* 2005;47(9):613-9.

42. Bower E, McLellan DL, Arney J, et al. A randomised controlled trial of different intensities of physiotherapy and different goal-setting procedures in 44 children with cerebral palsy. *Dev Med Child Neurol* 1996;38(3):226-37. doi: 10.1111/j.1469-8749.1996.tb15084.x

43. Lowing K, Bexelius A, Brogren Carlberg E. Activity focused and goal directed therapy for children with cerebral palsy--do goals make a difference? *Disabil Rehabil* 2009;31(22):1808-16. doi: 10.1080/09638280902822278

44. Lowing K, Bexelius A, Carlberg EB. Goal-directed functional therapy: a longitudinal study on gross motor function in children with cerebral palsy. *Disabil Rehabil* 2010;32(11):908-16. doi: 10.3109/09638280903353422

45. UNICEF. UNICEF 2017 Report on Communication for Development (C4D) 2017 [Available from: https://www.unicef.org/media/47781/file/UNICEF_2017_Report_on_Communication_for_Development_C4D.pdf.

46. Russell DJ, Avery LM, Rosenbaum PL, et al. Improved scaling of the gross motor function measure for children with cerebral palsy: evidence of reliability and validity. *Phys Ther* 2000;80(9):873-85. [published Online First: 2000/08/29]

47. Kakooza-Mwesige A, Tumwine JK, Forssberg H, et al. The Uganda version of the Pediatric Evaluation of Disability Inventory (PEDI). Part I: Cross-cultural adaptation. *Child Care Health Dev* 2018;44(4):552-61. doi: 10.1111/cch.12563 [published Online First: 2018/03/13]

48. Amer A, Kakooza-Mwesige A, Jarl G, et al. The Ugandan version of the Pediatric Evaluation of Disability Inventory (PEDI-UG). Part II: Psychometric properties. *Child Care Health Dev* 2018;44(4):562-71. doi: 10.1111/cch.12562 [published Online First: 2018/03/14]

49. Arvidsson P, Dada S, Granlund M, et al. Content validity and usefulness of Picture My Participation for measuring participation in children with and without intellectual disability in South Africa and Sweden. *Scand J Occup Ther* 2019:1-13. doi: 10.1080/11038128.2019.1645878

50. Demers L, Weiss-Lambrou R, Ska B. Development of the Quebec User Evaluation of Satisfaction with assistive Technology (QUEST). *Assist Technol* 1996;8(1):3-13. doi: 10.1080/10400435.1996.10132268 [published Online First: 1995/12/09]

51. Varni JW, Seid M, Kurtin PS. PedsQL 4.0: reliability and validity of the Pediatric Quality of Life Inventory version 4.0 generic core scales in healthy and patient populations. *Med Care* 2001;39(8):800-12.

52. Dambi JM, Tapera L, Chiwaridzo M, et al. Psychometric evaluation of the Shona version of the Multidimensional Scale of Perceived Social Support Scale (MSPSS-Shona) in adult informal caregivers of patients with cancer in Harare, Zimbabwe. *Malawi Med J* 2017;29(2):89-96. doi: 10.4314/mmj.v29i2.3

53. CBM. Disability Inclusive Development Toolkit 2017 [Available from: https://www.cbm.org/fileadmin/user_upload/Publications/CBM-DID-TOOLKIT-accessible.pdf.

For peer review only - http://bmjopen.bmj.com/site/about/guidelines.xhtml

54. Evans DB, Hsu J, Boerma T. Universal health coverage and universal access: World Health Organization; 2013 [Available from: http://dx.doi.org/10.2471/BLT.13.125450 [PDF].

For peer review only

Figure 1: Flow-chart for children in the Akwenda Cerebral Palsy Intervention Study

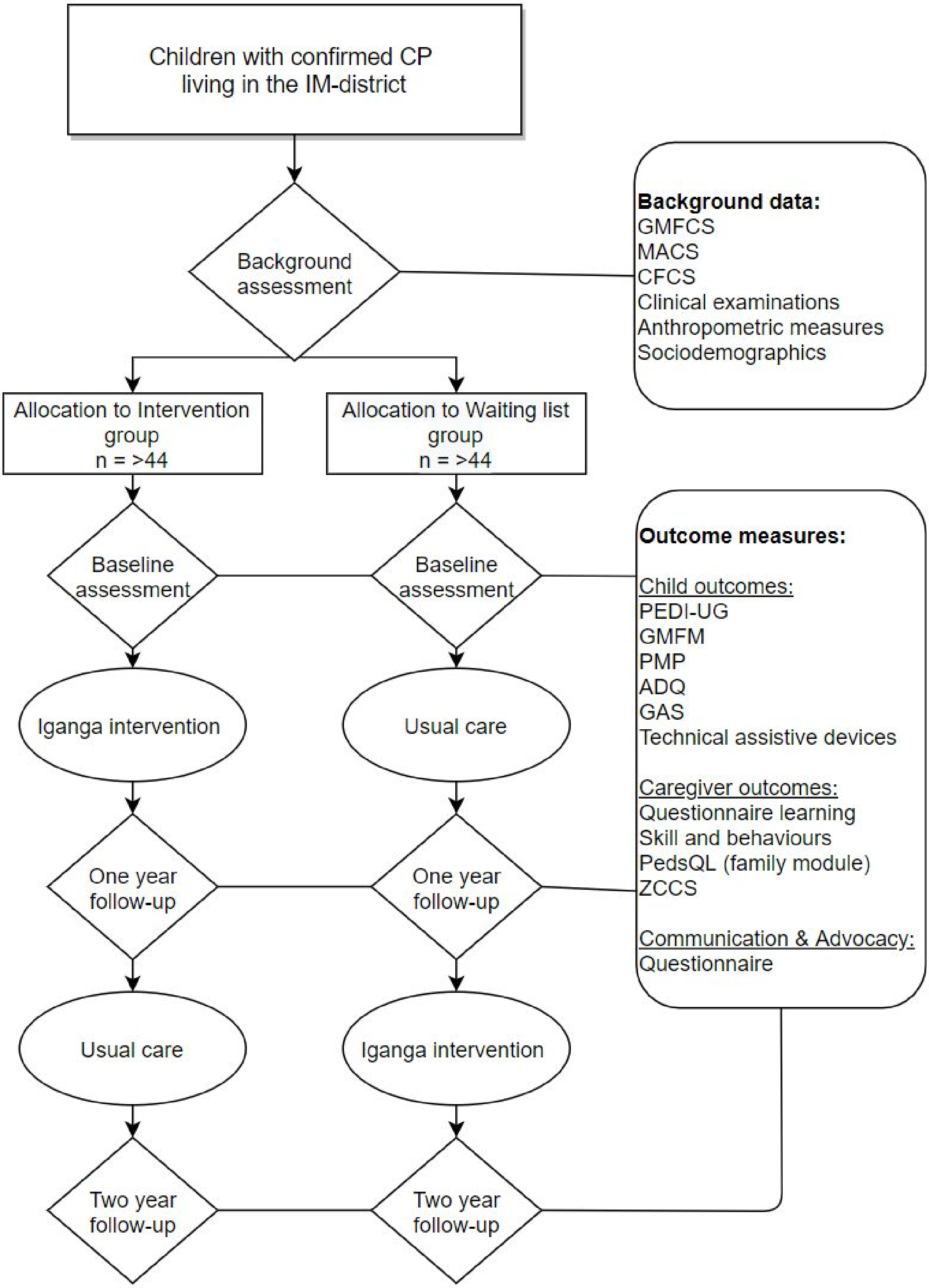

Activities over the 11 months of the Akwenda CP intervention programme

Components	1	2	3	4	5	6	7	8	9	10	11
**Caregiver-led training workshops**	What is CP?	CP as a way of life			Getting my child's body ready to be active	Eating & drinking	Communi-cation		Play	CVI	Recap
**Therapist-led practical group sessions and workshops**	What is CP?	CP as a way of life	Purpose use and maintenance of TAD	Setting realistic goals for my child	Getting my child's body ready to be active	Eating & drinking	Communi-cation	Nutrition & epilepsy	Play	CVI	Recap
**Goal setting**				Group workshop: refining the goals for my child	Home visit: Confirm goals & make individual action plan			Home visit: follow up goals & TAD			Review goals (GAS)
**Technical assistive devices (TAD)**	Decide on TAD for each child			Home visit: give out and fit TAD							
**Communication & Advocacy caregivers**	Rights of children with disabilities	Mapping of available services			Barriers to services for children with CP	Barriers to services for children with CP	Joint dialogue meeting: overcoming barriers and developing a community action plan				
**Communication & Advocacy stakeholders**		What is CP? Barriers to inclusion							Follow up of community action plan by community social worker		

Supplement 1:

The Akwenda cerebral palsy intervention programme

The Akwenda cerebral palsy (CP) programme was developed by an international team of experts within childhood disabilities and rehabilitation, an academic team from Uganda, a team from Malamulele Onward in South Africa, a team of therapists and a social worker working in Eastern Uganda, as well as four caregivers from the study area. Findings from our previous studies in the same cohort identifying the needs among the children and families, and the gaps in service provision also informed the process. The first step identified key principles based on current concepts of disability, rehabilitation and interventions for children with CP which would be possible to implement in low-resource settings. This involved reviewing literature and learning from programmes developed for low-resource settings with no or few health professionals. From this review,, the parent-led Carer-2-Carer Training Programme developed and implemented by Malamulele Onward in Johannesburg, South Africa was chosen to form the core of the Akwenda programme.[1] The second step involved clarifying the purpose of and agreeing on goals for the intervention programme. Based on the principles and goals formulated in previous steps, the five components of the intervention were defined in the third step.

Step 1: Key principles

The ICF framework

The first principle is the International Classification of Functioning and Disability and Health (ICF) framework which defines disability not as a characteristic of a person but as the interaction between health conditions and environmental and personal factors at the level of: i) body structure and function; ii) activity, and iii) participation in the family and society. The ICF has had an immense influence on changing perspectives of disability over the last decades in shifting the primary focus of interventions from impairments to participation and inclusion whilst emphasizing the need to address barriers, e.g., attitudinal, institutional and environmental.[2] The ICF framework has enabled community-based services to be brought closer to the children. It has also facilitated family oriented services, i.e. services that directly target the family's needs for information, education and psychosocial suppor.[3] The Akwenda CP programme has adopted the ICFs holistic view on disability whereby activity and participation are the main focuses of the programme. The programme also embraces the community-based and family oriented concept, through utilizing trained caregivers to train other caregivers, and by moving the services into the communities and homes of the participants.

For peer review only

Evidence based best practice

The second principle was to ensure that the Akwenda CP programme was based on evidence based best practice. A plethora of interventions are used today for children with CP, many without supportive scientific data.[4] The past decades have seen a surge of clinical trials resulting in a wider range of evidence based interventions, as well as interventions with strong evidence that they are not effective.[4] This emerging evidence shows that interventions improving functional ability in children with CP are child-active approaches where the child is actively practicing real-life tasks. Such child-active approaches are supported by current neuroscience demonstrating activity dependent learning induced plasticity in the brain underlying the learning and improvement of motor skills.[5] With little available information on evidence-based practices from LMIC, the activities of the Akwenda programme are therefore based on best clinical practice derived from clinical studies in HIC, adapted to the low income context in this rural part of Uganda. We have in addition also sought inspiration from programmes and studies done in LMIC which provide innovative strategies to provide the best care available given limited resources. [6 7]

Universal health coverage and universal access

The third principle is based on the WHO's goal of universal health coverage and universal access, which aims to ensure that all people obtain the health and rehabilitation services they need without fear of financial hardship.[8] To achieve universal health and rehabilitation coverage, services should be physically accessible, affordable and acceptable to patients. This means that services need to be available; located close to people; and should not entail financial hardship. Not only the expense of the health services but also the indirect and opportunity costs, e.g. transportation and time away from work need to be taken into account. Acceptability captures people's willingness to seek services, and depends on how patients perceive services and how social and cultural attitudes of the health provider encourage or discourage them from seeking services.[8] We have tried to ensure that the Akwenda CP programme is physically accessible, affordable and acceptable. Physical accessibility and affordability will be ensured through locating the activities in the community, utilising locally available resources for technical assistive devices, optimising the limited therapist time, and the utilization of trained caregivers. The programme also includes a communication and advocacy component aimed to ensure acceptability.

Step 2: Goals

Three general goals based on the principles and the needs of children and youth with cerebral palsy and their families, which had been identified in previous studies[9-11] were agreed upon:

- To improve motor function, activity and participation of the children and youth with cerebral palsy
- To increase knowledge and skill of the caregivers, reduce their stress and improve their mental health

- To facilitate children's inclusion and participation in society through increasing public awareness and reducing stigma in the community.

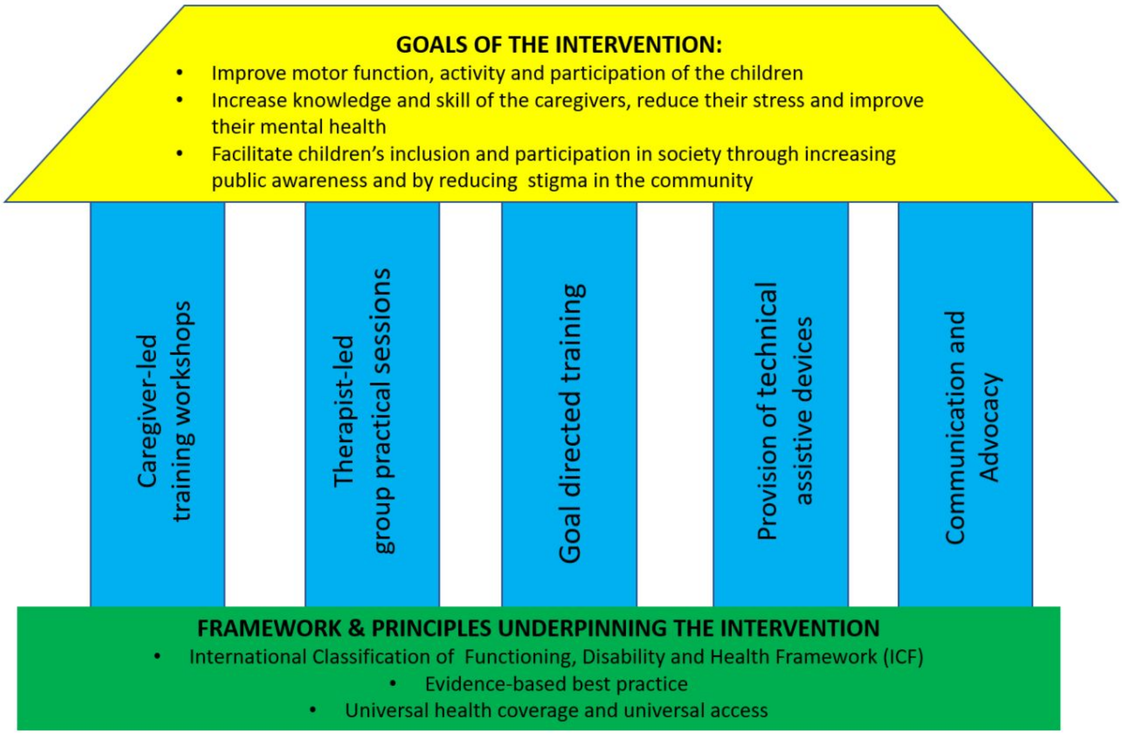

Figure 1: Illustration of the Akwenda CP programme: based on three key principles; comprising five intervention components; and aiming to achieve three goals.

Step 3: Five intervention components

Based on the principles and goals formulated in previous steps, the five components of the Akwenda CP programme are (See Figure1):

1. Caregiver-led training workshops
2. Therapist-led practical group sessions and workshops
3. Provision of technical assistive devices (TAD)
4. Goal setting
5. Communication and advocacy for behavioural and social change (C&A)

Component 1: Caregiver-led training workshops

The caregiver-led training workshops of the Malamulele Onward Carer-2-Carer Training Programme (MOC2CTP) form the cores of the Akwenda CP programme.[1 12] The MOC2CTP trains primary caregivers of children with CP to be Parent Facilitators, able to organise and lead seven training workshops (Table 1). The MOC2CTP was developed and refined over a

seven-year period by a multidisciplinary team of three therapists. The original group of 20 Parent Facilitators themselves were major contributors to the final product through their feedback and experiences in running the programme.

	Title of workshop	Outcomes
1	What is CP and how does it affect my child?	• Be able to explain what CP is to a family member<br>• Know the GMFCS Level the child falls into<br>• Know what kind (subtype) of CP the child has<br>• Understand the possible causes of CP, and what does not cause CP, especially that CP is not a disease and not her fault
2	CP as a way of life	• What does "Caring for child with CP is a way of life" mean<br>• How children with CP change over time<br>• How we can do everyday things in a way that is helpful to children with CP<br>• Helpful ways to hold and move children with CP<br>• Helpful ways to put children with CP in different positions
3	Getting my child's body ready to move and be active	• How to get a child's body ready to move and play<br>• How to position a child in a helpful way<br>• How to use these ideas in everyday activities
4	Eating and drinking: making mealtimes safe and comfortable for my child	• How to best position a child with CP for feeding<br>• Why it is important to feed a child with CP slowly<br>• How to prepare the child's lips and mouth for eating<br>• How to help a child with CP to eat better with a spoon<br>• How to help a child with CP to learn to chew<br>• How to help a child feed himself<br>• How to help a child with CP learn to drink from a cup<br>• How to help children who drool
5	Communication: my child and I understanding each other	• What we mean by communication<br>• The different ways we can communicate<br>• How to encourage a child with CP to communicate<br>• How to help a child with CP to understand better<br>• How to help a child with CP to talk better<br>• Helping children to use signs
6	Play: unlocking my child's potential	• What is play and why it is important for a child with CP<br>• How children usually play<br>• Why it is difficult for children with CP to play<br>• How we can make it easier for all children with CP to play<br>• What we can play with
7	CVI: Understanding where and what my child can see	• Understand vision difficulties<br>• What does CVI stand for and what does it mean<br>• How to know if a child has a CVI<br>• How to play with a child with a CVI<br>• How we use what the child sees best in everyday life

Table 1: Parent led workshops based on Malamulele Onward Carer-2-Carer Training Programme (each workshop runs for 2 – 2 ½ hours).

To implement the Akwenda CP programme, four caregivers (three mothers and one aunt) from Iganga were identified based on their availability, motivation, initiative, interest, and

For peer review only

communication skills. They received four weeks training from a master trainer from Malamulele Onward, herself a parent of a child with CP.

Each of the seven trainings is approximately two hours long and will be led by two Iganga Parent Facilitators working as a team. To ensure quality and consistency of the workshops, the Parent Facilitators will follow the detailed Malamulele Onward Carer-2-Carer Training Programme manual which has been translated into Lusoga, the local language. For each workshop the Parent Facilitators have a workshop kit, which includes display materials. No therapist will be involved in these seven caregiver-led workshops.

Component 2: Therapist-led practical group sessions and workshops

Two weeks following each caregiver-led workshop, caregivers and children will attend a therapist-led practical group sessions that will have the same themes as the caregiver-led training workshop (Table 1). The therapists will be assisted by the Parent Facilitators during these practical sessions. Active parent participation is an essential feature of the group practical sessions which will be structured in such a way that the therapist will be able to give each child individual attention during the group activities.

In addition, the therapists involved in the intervention will run four group training workshops on: i) technical assistive devices; ii) goal directed training and setting realistic goals; iii) nutrition and iv) epilepsy (Table 2). In the previous study on this cohort of children with CP we noted that almost half (46%) of the children were severely malnourished, and 35% of the children suffered from seizures.[13] A manual for these sessions have been developed to ensure quality and consistency.

	Title of workshop	Outcomes
1	Healthy diet and healthy eating	• Know the seven major classes of nutrients<br>• Know how to prepare a meal from locally available nutrient dense foods<br>• Understand responsive feeding practices (e.g. frequency of feeding; creating a safe and clean environment for feeding)
2	Understanding epilepsy	• How to recognise a seizure<br>• Understand misconceptions related to epilepsy and how to deal with stigma<br>• Know when and where to seek care for a child with seizures<br>• Understand the role of traditional healers and traditional treatments<br>• Understand anti-epileptic medicine – how to administer; side effects and the importance of adherence

Table 2: Therapist-led training workshops on nutrition and seizures (each workshop is one hour long)

For peer review only - http://bmjopen.bmj.com/site/about/guidelines.xhtml

The two therapists running these practical sessions are experienced in working with children with CP in rural areas of Uganda, and have received 12 days of training on the practical management of children with CP. This training included essential concepts and components of the intervention programme viz., functional activities; the child's active participation and the value of self-generated movements; goal setting; working in groups; working in partnership with parents; technical assistive devices; nutrition and epilepsy. A therapist manual detailing the structure and content of each practical session has been drawn up for the intervention.

Component 3: Provision of technical assistive devices (TAD)

Provision of technical assistive devices (TAD) is an important part of evidence based best practice as they promote greater independence and enables children to perform tasks they were formerly unable to accomplish within the domains of mobility, self-care, eating and communication.[14] In this study, the aim of provision of TAD will be to simplify caregiving and increase the child's activity and participation.

The therapists will together with the caregivers and children assess the needs of TAD during the baseline assessments. Children and youth with lower limb contractures who would benefit from the provision of a standing frame or walking device will be referred to the local hospital for serial casting if it is thought that serial casting will provide sufficient soft tissue length to enable the child or youth to stand in alignment and with their feet in an acceptable position.

Where possible, TAD will be locally manufactured with affordable local materials, and the caregiver will be involved in cost sharing to ensure affordability and sustainability. Postural mobility devices, e.g., wheelchairs or buggies are costly and difficult to make locally, and will be procured from the Ministry of Health's accredited assistive devices production unit and/or donor agencies.

Children at GMFCS III-IV will be provided with standing frames, customised posture support chairs, walking assistive device and parallel bars f all of which will be made for use in the home environment by local artisans from either wood or using appropriate paper technology. Simple communication books or boards will be provided for children with communication difficulties. Children and youth will be issued with re-usable nappies or sanitary pads if needed. Other devices will be procured or created by the treating therapist in collaboration with the primary caregiver as the need arises, such as the construction of an accessible toilet seat, cut out feeding cups, shoes, splints, and appropriate toys.

The TAD will be issued during the 3rd month of the intervention following a therapist-led training workshop explaining the use, maintenance and care of assistive devices. The therapist together with the community social worker will deliver the TAD to the child's home where they will also ensure correct fitting of the device. Usage of the TAD will be evaluated during a home visit in the 8th month.

For peer review only

Component 4: Goal setting

Goal directed therapy is a child-active, motor learning approach, and includes daily practice directly targeting the achievement of a goal.[15] The child and family, supported by health workers, identify realistic goals and create action plans for how to achieve them. Several studies have demonstrated the success of goal directed programs in HIC,[16-19] while there are no reports from LMIC. The goal-setting procedure in the Akwenda CP programme will start during the fourth month when the therapist has studied the assessment results of the child and become acquainted with the child and caregiver and the caregiver has attended the first two caregiver-led training workshops, meaning that she/he has a better understanding of the child's condition and potential. The therapist will lead two consecutive group sessions to explain the concept of goal setting to parents, during which the parents will assist one another in identifying goal-areas for their child. Following the group sessions, the therapists will visit each child in their home setting where the child and caregiver supported by the therapist will set three goals based on the goal-areas identified by the parents. An individualised activity plan will thereafter be made for each child, with the aim of achieving the goals within five months through daily training and practice in the home environment. Assessments performed before the intervention begins, including the Gross Motor Function Measurement (GMFM),[20] the Ugandan version of the Pediatric Evaluation of Disability Inventory (PEDI-UG), [21 22] and Picture my Participation measure,[23] will be used to inform the goal-setting procedure by identifying realistic goals based on the child's abilities and potential. These assessments explore different dimensions within the ICF framework, i.e., functional limitations, ability to perform activities and participation.

Each of the three goals will be scaled by the therapist as goal attainment scale (GAS) goals. For children issued with TAD, a fourth GAS goal will relate to the use of these TAD. Three months later, all GAS goals and the individualised activity plans for each child will be reviewed during the monthly group session or at a home visit; and re-evaluated in the 11th month.

Component 5:
Communication and Advocacy for behavioural and social change (C&A)

This component is based on Communication for Development (C4D), a systematic, planned and evidence-based strategy to promote positive social and behavioural changes.[24] It addresses the stigma, discrimination and exclusion of children with disability and forms an important part in making the Akwenda cerebral palsy programme accessible and acceptable. Target groups include community leaders, healthcare professionals, school educators, the general population, families, and the caregivers themselves.

Caregivers of the children with CP in the intervention group will participate in three C&A meetings to be conducted in months one, two, and six of the intervention. Each meeting will

For peer review only - http://bmjopen.bmj.com/site/about/guidelines.xhtml

last 90 minutes and be led by the community social worker. It will take place on the same day as one of the parent-led workshops. Topics for these meetings are (i) rights of children with disabilities with a focus on human rights and abuses of children with CP in the community; (ii) mapping available health, education and legal services in the community; and (iii) barriers to services and how to overcome those barriers. These discussions and the proposed solutions will then be discussed with the community leaders at a joint dialogue session the 7th month.

Forty community representatives, including political, administrative, cultural and religious leaders, educators, health workers, police, disability NGO/CBO representatives, district and sub-county technical personnel will be invited to participate in two C&A meetings led by the community social worker. The first session will focus on the rights of children with disabilities and the stigma they meet leading to discrimination and barriers limiting their participation. The second will be a joint session with the caregivers discussing how to make services inclusive and to develop a community action plan. The community social worker will follow up the implementation of the community action plan in months nine to eleven of the intervention through regular telephone feedback from participants and/or visits to the participating communities.

In preparation for the C & A component and to sensitize the community to disability issues, information on cerebral palsy will be delivered through a one-hour talk show on six community radio stations by the community social worker. Messages will include information relating to how the CURIE III study intends to assist children with cerebral palsy in Iganga-Mayuge. A telephone number of the community social worker will be shared with the listeners at the end of each radio talk show.

Time table and delivery of the integrated intervention

The time table for delivering the intervention through the five activities is shown in Table 1. It will be delivered over a period of 11 months with caregivers attending two sessions per month, one of which will be together with the child. In addition, there will be three home visits and five C&A sessions. Two therapists, four parent facilitators and one community social worker will deliver the services required.

Discussion

The aim of the Akwenda CP intervention programme for children and youth with CP is to provide a comprehensive spectrum of interventions for the child, the caregiver and the community. The core of the intervention is a carer-to-carer training programme. Parent training programmes have previously been reported from Ghana and West Bengal, [6 7] however, that training was either delivered by health workers or community disability workers, only some of whom were mothers of children with disabilities.

For peer review only

In order to make this intervention holistic and comprehensive to impact the child, the caregiver and the wider community, the carer-to-carer training has been complemented with additional activities aiming to improve children's functional abilities, activities and participation as well as a community and advocacy programme aiming at social and behavioural changes in family and community. Good clinical practice should strive to address the various ICF domains at the same time since improvements in one domain may not transfer directly to others.[25]

In line with WHO's goal of universal health coverage, we have tried to make the Akwenda CP programme physically accessible, affordable and acceptable. The venue for the intervention is a community setting within easy reach of the children and caregivers, making it physically accessible in a timely manner with minimal transportation costs. The intervention is designed to be affordable for caregivers living in a low resource setting where many are living at or below the poverty level, and with a scarcity of therapists and other rehabilitation professionals. Therapists are essential but their skills and time need to be optimised and leveraged, and the use of caregivers as in this programme, is one way to achieve this. TAD are largely locally manufactured.

Stigma and discrimination increases the risk that children with CP are neglected by primary health care and education services. The communication and advocacy component aims at social and behavioural change in the local community but particularly targeting health workers, teachers and community leaders. Empowerment of children and parents will increase their confidence and emphasize their rights to receive basic health and rehabilitation services. Working with caregivers and children in group settings, as opposed to working on an individual one to one setting, will also unify parents in similar situations and strengthen their confidence.

Currently, there are few evidence-based interventions that have been evaluated in LMICs. The activities of the Akwenda CP intervention programme is therefore based on best clinical practice derived from clinical studies in HIC, and adapted to the conditions in this rural part of Uganda in accordance with the WHO's goal of universal health coverage and universal access.[8] The multiple activities add to the complexity of the intervention as well as its evaluation. This study will provide information on the affordability and cost-benefit ratio of the provision of a comprehensive intervention in low-resource setting.

References:

1. Onward M. CARER-2-CARER TRAINING PROGRAMME 2020 [Available from: https://www.malamuleleonward.org/what-we-offer/carer-2-carer-programme.
2. World Health Organization. International classification of functioning, disability and health: children and youth version: ICF-CY 2007 [Available from: https://apps.who.int/iris/handle/10665/43737.

3. King G, Williams L, Goldberg SH. Family-oriented services in pediatric rehabilitation: a scoping review and framework to promote parent and family wellness. *Child Care Hlth Dev* 2017;43(3):334-47. doi: 10.1111/cch.12435
4. Novak I, McIntyre S, Morgan C, et al. A systematic review of interventions for children with cerebral palsy: state of the evidence. *Dev Med Child Neurol* 2013;55(10):885-910. doi: 10.1111/dmcn.12246
5. Diaz Heijtz R, Forssberg H. Translational studies exploring neuroplasticity associated with motor skill learning and the regulatory role of the dopamine system. *Dev Med Child Neurol* 2015;57 Suppl 2:10-4. doi: 10.1111/dmcn.12692
6. Benfer KA, Novak I, Morgan C, et al. Community-based parent-delivered early detection and intervention programme for infants at high risk of cerebral palsy in a low-resource country (Learning through Everyday Activities with Parents (LEAP-CP): protocol for a randomised controlled trial. *BMJ Open* 2018;8(6):e021186. doi: 10.1136/bmjopen-2017-021186
7. Zuurmond M, Nyante G, Baltussen M, et al. A support programme for caregivers of children with disabilities in Ghana: Understanding the impact on the wellbeing of caregivers. *Child Care Health Dev* 2019;45(1):45-53. doi: 10.1111/cch.12618
8. Evans DB, Hsu J, Boerma T. Universal health coverage and universal access: World Health Organization; 2013 [Available from: http://dx.doi.org/10.2471/BLT.13.125450 [PDF].
9. Kakooza-Mwesige A, Andrews C, Peterson S, et al. Prevalence of cerebral palsy in Uganda: a population-based study. *Lancet Glob Health* 2017;5(12):e1275-e82. doi: 10.1016/S2214-109X(17)30374-1
10. Andrews C, Kakooza-Mwesige A, Almeida R, et al. Impairments, functional limitations, and access to services and education for children with cerebral palsy in Uganda: a population-based study. *Dev Med Child Neurol* 2019 doi: 10.1111/dmcn.14401
11. Kakooza-Mwesige A, Tumwine JK, Eliasson AC, et al. Malnutrition is common in Ugandan children with cerebral palsy, particularly those over the age of five and those who had neonatal complications. *Acta Paediatr* 2015;104(12):1259-68. doi: 10.1111/apa.13089
12. Saloojee G, Bezuidenhout M. Parent-led services and community-based peer supporters for children and adults with disabilities: experiences from two programmes. In: Padarath A, Kathard H, Lorenzo T, et al., eds. South African Health Review 2020. Durban: Health Systems Trust 2020.
13. Andrews C, Kakooza-Mwesige A, Almeida R, et al. Impairments, functional limitations, and access to services and education for children with cerebral palsy in Uganda: a population-based study. *Dev Med Child Neurol* 2020;62(4):454-62. doi: 10.1111/dmcn.14401
14. WHO. Global Cooperation on Assistive Technology (GATE) 2020 [Available from: https://www.who.int/disabilities/technology/gate/en/.
15. Ketelaar M, Vermeer A, Hart H, et al. Effects of a functional therapy program on motor abilities of children with cerebral palsy. *Phys Ther* 2001;81(9):1534-45. doi: 10.1093/ptj/81.9.1534
16. Ahl LE, Johansson E, Granat T, et al. Functional therapy for children with cerebral palsy: an ecological approach. *Dev Med Child Neurol* 2005;47(9):613-9.
17. Bower E, McLellan DL, Arney J, et al. A randomised controlled trial of different intensities of physiotherapy and different goal-setting procedures in 44 children with cerebral palsy. *Dev Med Child Neurol* 1996;38(3):226-37. doi: 10.1111/j.1469-8749.1996.tb15084.x

For peer review only

18. Lowing K, Bexelius A, Brogren Carlberg E. Activity focused and goal directed therapy for children with cerebral palsy--do goals make a difference? *Disabil Rehabil* 2009;31(22):1808-16. doi: 10.1080/09638280902822278
19. Lowing K, Bexelius A, Carlberg EB. Goal-directed functional therapy: a longitudinal study on gross motor function in children with cerebral palsy. *Disabil Rehabil* 2010;32(11):908-16. doi: 10.3109/09638280903353422
20. Russell DJ, Avery LM, Rosenbaum PL, et al. Improved scaling of the gross motor function measure for children with cerebral palsy: evidence of reliability and validity. *Phys Ther* 2000;80(9):873-85.
21. Kakooza-Mwesige A, Tumwine JK, Forssberg H, et al. The Uganda version of the Pediatric Evaluation of Disability Inventory (PEDI). Part I: Cross-cultural adaptation. *Child Care Health Dev* 2018;44(4):552-61. doi: 10.1111/cch.12563 [published Online First: 2018/03/13]
22. Amer A, Kakooza-Mwesige A, Jarl G, et al. The Ugandan version of the Pediatric Evaluation of Disability Inventory (PEDI-UG). Part II: Psychometric properties. *Child Care Health Dev* 2018;44(4):562-71. doi: 10.1111/cch.12562 [published Online First: 2018/03/14]
23. Arvidsson P, Dada S, Granlund M, et al. Content validity and usefulness of Picture My Participation for measuring participation in children with and without intellectual disability in South Africa and Sweden. *Scand J Occup Ther* 2019:1-13. doi: 10.1080/11038128.2019.1645878
24. UNICEF. UNICEF 2017 Report on Communication for Development (C4D) 2017 [Available from: https://www.unicef.org/media/47781/file/UNICEF_2017_Report_on_Communication_for_Development_C4D.pdf.
25. Novak I, Morgan C, Fahey M, et al. State of the Evidence Traffic Lights 2019: Systematic Review of Interventions for Preventing and Treating Children with Cerebral Palsy. *Curr Neurol Neurosci Rep* 2020;20(2):3. doi: 10.1007/s11910-020-1022-z

For peer review only

STANDARD PROTOCOL ITEMS: RECOMMENDATIONS FOR INTERVENTIONAL TRIALS

SPIRIT 2013 Checklist: Recommended items to address in a clinical trial protocol and related documents*

Section/item	Item No	Description	Addressed on page number
**Administrative information**			
Title	1	Descriptive title identifying the study design, population, interventions, and, if applicable, trial acronym	______1______
Trial registration	2a	Trial identifier and registry name. If not yet registered, name of intended registry	______2 & 11__
	2b	All items from the World Health Organization Trial Registration Data Set	______11______
Protocol version	3	Date and version identifier	______?_______
Funding	4	Sources and types of financial, material, and other support	_____14_______
Roles and responsibilities	5a	Names, affiliations, and roles of protocol contributors	____1 and 13___
	5b	Name and contact information for the trial sponsor	______14_____
	5c	Role of study sponsor and funders, if any, in study design; collection, management, analysis, and interpretation of data; writing of the report; and the decision to submit the report for publication, including whether they will have ultimate authority over any of these activities	______14_____
	5d	Composition, roles, and responsibilities of the coordinating centre, steering committee, endpoint adjudication committee, data management team, and other individuals or groups overseeing the trial, if applicable (see Item 21a for data monitoring committee)	______N/A______

For peer review only - http://bmjopen.bmj.com/site/about/guidelines.xhtml

Introduction

Background and rationale	6a	Description of research question and justification for undertaking the trial, including summary of relevant studies (published and unpublished) examining benefits and harms for each intervention	____4-5______
	6b	Explanation for choice of comparators	_not applicable___
Objectives	7	Specific objectives or hypotheses	_____5-6_______
Trial design	8	Description of trial design including type of trial (eg, parallel group, crossover, factorial, single group), allocation ratio, and framework (eg, superiority, equivalence, noninferiority, exploratory)	_____6____

Methods: Participants, interventions, and outcomes

Study setting	9	Description of study settings (eg, community clinic, academic hospital) and list of countries where data will be collected. Reference to where list of study sites can be obtained	______6____
Eligibility criteria	10	Inclusion and exclusion criteria for participants. If applicable, eligibility criteria for study centres and individuals who will perform the interventions (eg, surgeons, psychotherapists)	______6
Interventions	11a	Interventions for each group with sufficient detail to allow replication, including how and when they will be administered	7 - 8 + Suppl 1
	11b	Criteria for discontinuing or modifying allocated interventions for a given trial participant (eg, drug dose change in response to harms, participant request, or improving/worsening disease)	__not applicable
	11c	Strategies to improve adherence to intervention protocols, and any procedures for monitoring adherence (eg, drug tablet return, laboratory tests)	_Suppl 1_
	11d	Relevant concomitant care and interventions that are permitted or prohibited during the trial	______8_______
Outcomes	12	Primary, secondary, and other outcomes, including the specific measurement variable (eg, systolic blood pressure), analysis metric (eg, change from baseline, final value, time to event), method of aggregation (eg, median, proportion), and time point for each outcome. Explanation of the clinical relevance of chosen efficacy and harm outcomes is strongly recommended	_____9 - 10____
Participant timeline	13	Time schedule of enrolment, interventions (including any run-ins and washouts), assessments, and visits for participants. A schematic diagram is highly recommended (see Figure)	6 + Suppl 1 and Table 1

For peer review only - http://bmjopen.bmj.com/site/about/guidelines.xhtml

Sample size	14	Estimated number of participants needed to achieve study objectives and how it was determined, including clinical and statistical assumptions supporting any sample size calculations	______8_____
Recruitment	15	Strategies for achieving adequate participant enrolment to reach target sample size	______8_____

Methods: Assignment of interventions (for controlled trials)

Allocation:

Sequence generation	16a	Method of generating the allocation sequence (eg, computer-generated random numbers), and list of any factors for stratification. To reduce predictability of a random sequence, details of any planned restriction (eg, blocking) should be provided in a separate document that is unavailable to those who enrol participants or assign interventions	______7_____
Allocation concealment mechanism	16b	Mechanism of implementing the allocation sequence (eg, central telephone; sequentially numbered, opaque, sealed envelopes), describing any steps to conceal the sequence until interventions are assigned	______7_______
Implementation	16c	Who will generate the allocation sequence, who will enrol participants, and who will assign participants to interventions	_______7_____
Blinding (masking)	17a	Who will be blinded after assignment to interventions (eg, trial participants, care providers, outcome assessors, data analysts), and how	_______7_____
	17b	If blinded, circumstances under which unblinding is permissible, and procedure for revealing a participant's allocated intervention during the trial	__not applicable

Methods: Data collection, management, and analysis

Data collection methods	18a	Plans for assessment and collection of outcome, baseline, and other trial data, including any related processes to promote data quality (eg, duplicate measurements, training of assessors) and a description of study instruments (eg, questionnaires, laboratory tests) along with their reliability and validity, if known. Reference to where data collection forms can be found, if not in the protocol	_______9-10___
	18b	Plans to promote participant retention and complete follow-up, including list of any outcome data to be collected for participants who discontinue or deviate from intervention protocols	______?______

Data management	19	Plans for data entry, coding, security, and storage, including any related processes to promote data quality (eg, double data entry; range checks for data values). Reference to where details of data management procedures can be found, if not in the protocol	_____10-11_____
Statistical methods	20a	Statistical methods for analysing primary and secondary outcomes. Reference to where other details of the statistical analysis plan can be found, if not in the protocol	______11_______
	20b	Methods for any additional analyses (eg, subgroup and adjusted analyses)	______11_______
	20c	Definition of analysis population relating to protocol non-adherence (eg, as randomised analysis), and any statistical methods to handle missing data (eg, multiple imputation)	______?______
**Methods: Monitoring**			
Data monitoring	21a	Composition of data monitoring committee (DMC); summary of its role and reporting structure; statement of whether it is independent from the sponsor and competing interests; and reference to where further details about its charter can be found, if not in the protocol. Alternatively, an explanation of why a DMC is not needed	___11-12_______
	21b	Description of any interim analyses and stopping guidelines, including who will have access to these interim results and make the final decision to terminate the trial	______12_______
Harms	22	Plans for collecting, assessing, reporting, and managing solicited and spontaneously reported adverse events and other unintended effects of trial interventions or trial conduct	______12_______
Auditing	23	Frequency and procedures for auditing trial conduct, if any, and whether the process will be independent from investigators and the sponsor	__not applicable__
**Ethics and dissemination**			
Research ethics approval	24	Plans for seeking research ethics committee/institutional review board (REC/IRB) approval	______11_____
Protocol amendments	25	Plans for communicating important protocol modifications (eg, changes to eligibility criteria, outcomes, analyses) to relevant parties (eg, investigators, REC/IRBs, trial participants, trial registries, journals, regulators)	________11__

Consent or assent	26a	Who will obtain informed consent or assent from potential trial participants or authorised surrogates, and how (see Item 32)	______ 11_____
	26b	Additional consent provisions for collection and use of participant data and biological specimens in ancillary studies, if applicable	_not applicable___
Confidentiality	27	How personal information about potential and enrolled participants will be collected, shared, and maintained in order to protect confidentiality before, during, and after the trial	_____11_______
Declaration of interests	28	Financial and other competing interests for principal investigators for the overall trial and each study site	_______14_____
Access to data	29	Statement of who will have access to the final trial dataset, and disclosure of contractual agreements that limit such access for investigators	________14______
Ancillary and post-trial care	30	Provisions, if any, for ancillary and post-trial care, and for compensation to those who suffer harm from trial participation	_not applicable_
Dissemination policy	31a	Plans for investigators and sponsor to communicate trial results to participants, healthcare professionals, the public, and other relevant groups (eg, via publication, reporting in results databases, or other data sharing arrangements), including any publication restrictions	________12____
	31b	Authorship eligibility guidelines and any intended use of professional writers	__not applicable__
	31c	Plans, if any, for granting public access to the full protocol, participant-level dataset, and statistical code	________14______
**Appendices**			
Informed consent materials	32	Model consent form and other related documentation given to participants and authorised surrogates	__ Supplement___
Biological specimens	33	Plans for collection, laboratory evaluation, and storage of biological specimens for genetic or molecular analysis in the current trial and for future use in ancillary studies, if applicable	__not applicable

*It is strongly recommended that this checklist be read in conjunction with the SPIRIT 2013 Explanation & Elaboration for important clarification on the items. Amendments to the protocol should be tracked and dated. The SPIRIT checklist is copyrighted by the SPIRIT Group under the Creative Commons "Attribution-NonCommercial-NoDerivs 3.0 Unported" license.

For peer review only - http://bmjopen.bmj.com/site/about/guidelines.xhtml

BMJ Open

Akwenda intervention programme for children and youth with cerebral palsy in a low-resource setting in sub-Saharan Africa: protocol for a quasi-randomised controlled study

Journal:	*BMJ Open*
Manuscript ID	bmjopen-2020-047634.R1
Article Type:	Protocol
Date Submitted by the Author:	09-Feb-2021

Complete List of Authors:	Saloojee, Gillian; Independent researcher<br>Ekwan, Francis; Mulago National Referral Hospital, Department of Occupational Therapy,<br>Andrews, Carin; Karolinska Institutet, Department of Women's and Children's Health<br>Damiano, Diane; The National Institutes of Health Clinical Cente, Rehabilitation Medicine Department<br>Kakooza, Angelina; Mulago Hospital/Makerere University, Department of Paediatrics and Child Health<br>Forssberg, Hans; Karolinska Institutet, Women's and Children's Health

**Primary Subject Heading**:	Paediatrics
Secondary Subject Heading:	Rehabilitation medicine
Keywords:	Developmental neurology & neurodisability < PAEDIATRICS, REHABILITATION MEDICINE, Community child health < PAEDIATRICS

SCHOLARONE™
Manuscripts

I, the Submitting Author has the right to grant and does grant on behalf of all authors of the Work (as defined in the below author licence), an exclusive licence and/or a non-exclusive licence for contributions from authors who are: i) UK Crown employees; ii) where BMJ has agreed a CC-BY licence shall apply, and/or iii) in accordance with the terms applicable for US Federal Government officers or employees acting as part of their official duties; on a worldwide, perpetual, irrevocable, royalty-free basis to BMJ Publishing Group Ltd ("BMJ") its licensees and where the relevant Journal is co-owned by BMJ to the co-owners of the Journal, to publish the Work in this journal and any other BMJ products and to exploit all rights, as set out in our licence.

The Submitting Author accepts and understands that any supply made under these terms is made by BMJ to the Submitting Author unless you are acting as an employee on behalf of your employer or a postgraduate student of an affiliated institution which is paying any applicable article publishing charge ("APC") for Open Access articles. Where the Submitting Author wishes to make the Work available on an Open Access basis (and intends to pay the relevant APC), the terms of reuse of such Open Access shall be governed by a Creative Commons licence – details of these licences and which Creative Commons licence will apply to this Work are set out in our licence referred to above.

Other than as permitted in any relevant BMJ Author's Self Archiving Policies, I confirm this Work has not been accepted for publication elsewhere, is not being considered for publication elsewhere and does not duplicate material already published. I confirm all authors consent to publication of this Work and authorise the granting of this licence.

For peer review only

Title:

Akwenda intervention programme for children and youth with cerebral palsy in a low-resource setting in sub-Saharan Africa: protocol for a quasi-randomised controlled study.

Authors:

Gillian Saloojee, Independent researcher, Johannesburg, South Africa
E-mail: gillian.saloojee@gmail.com

Francis Ekwan, Principal Occupational Therapist, Department of Occupational Therapy, Mulago National Referral Hospital, Kampala, Uganda
E-mail: ekwanfr@yahoo.com

Carin Andrews, Neuropaediatrics, Department of Women's and Children's Health, Karolinska Institutet, Stockholm, Sweden
E-mail: carin.andrews@ki.se

Diane Damiano, National Institutes of Health, Bethesda, MD, USA.
E-mail: damianod@cc.nih.gov

Angelina Kakooza-Mwesige, Department of Paediatrics and Child Health, Makerere University College of Health Sciences, Kampala, Uganda
E-mail: angelina_kakooza@yahoo.co.uk

Hans Forssberg, Neuropaediatrics, Department of Women's and Children's Health, Karolinska Institutet, Stockholm, Sweden
E-mail: hans.forssberg@ki.se

Corresponding author:

Hans Forssberg, Neuropaediatrics, Department of Women's and Children's Health, Astrid Lindgren Children's Hospital, Karolinska Institutet, 1776 Stockholm, Sweden
E-mail: hans.forssberg@ki.se
Phone: +46 708 730830

Keywords:
Cerebral palsy, community based intervention, family-oriented services, communication and advocacy, low and middle-income country

Word count:
Abstract: 300 words
Main article: 3 983 words

For peer review only

Abstract

Introduction

Cerebral palsy (CP) is the most common childhood-onset motor disorder accompanied by associated impairments placing a heavy burden on families and health systems. Most children with CP live in low- and middle-income countries (LMICs) with little access to rehabilitation services. This study will evaluate the Akwenda CP programme, a multi-dimensional intervention designed for low resource settings and aiming at improving: i) participation, motor function and daily activities for children with CP; ii) quality of life, stress and knowledge for caregivers; and iii) knowledge and attitudes towards children with CP in the communities.

Methods

This quasi-randomized controlled, clinical study will recruit children and youth with CP aged 2-23 in a rural area of Uganda. Children will be allocated to one of two groups with at least 44 children in each group. Groups will be matched for age, sex and motor impairment. The intervention arm will receive a comprehensive, multi-dimensional programme over a period of 11 months comprising i) caregiver-led training workshops, ii) therapist-led practical group sessions, iii) provision of technical assistive devices, iv) goal directed training and v) community communication and advocacy. The other group will receive usual care. The outcome of the intervention will be assessed before and after the intervention and will be measured at three levels, (i) child, ii) caregiver, and iii) community. Standard analysis methods for randomised controlled trial will be used to compare groups. Retention of effects will be examined at 12-month follow-up.

Ethics and dissemination

The study has been approved by the Uganda National Council for Science and Technology (UNCST) (SS 5173) and registered in accordance with WHO and ICMJE standards at the Pan African Clinical Trials Registry (PACTR202011738099314). Written informed consent will be obtained from caregivers. Results will be disseminated among participants and stakeholders through public engagement events, scientific reports and conference presentations.

For peer review only

For peer review only - http://bmjopen.bmj.com/site/about/guidelines.xhtml

Strengths and limitations of this study

- This is the first registered randomized controlled clinical trial of a comprehensive, multi-dimensional intervention programme for children over the age of two years and youth with CP in a low and middle-income country.
- The core of the programme is based on principles derived from the International Classification of Functioning, Disability and Health (ICF), evidence based interventions and universal health coverage.
- The study will address several ICF categories and include a battery of objective, validated and adapted outcome measures that will measure a broad spectrum of intervention outcomes at the level of the child, the caregiver and the community.
- The small sample size will make it difficult to identify small effects and it will not be possible to identify which of the components is most effective.

For peer review only

INTRODUCTION

Cerebral palsy (CP) is a life-long motor disorder due to non-progressive disturbances of the developing foetal or infant brain. [1] In addition to gross and fine motor impairments, children with CP often have associated impairments in hearing, vision, cognition and communication, as well as behavioural problems and seizures. Feeding difficulties result in a high prevalence of malnutrition. [2] Apart from access to mainstream health care, social services and education, children with CP require services for their special needs. These needs are not met in many low- and middle-income countries (LMICs) where children with developmental disabilities often are neglected by primary health care and education services and face discrimination and exclusion.[3 4] Discrimination often stems from a lack of knowledge, prejudice and cultural norms, which in turn lead to stigma and entrenched social exclusion.[5-7] LMICs are characterised by a paucity of health professionals specialized in childhood disability including physio-, occupational- and speech and language therapists,[8, 9 10] as well as a lack of assistive devices.[11] Professional consensus reports from Africa illustrate an immense lack of services for children with neurodevelopmental disorders with limited health worker and caregiver awareness, poor community attitudes and practices, lack of health worker training with unclear treatment or referral guidelines and a lack of diagnostic facilities or feasible health interventions.[12-14]

The scarcity of rehabilitation services has resulted in families being left alone to care for the child with CP, neither knowing the cause and the life-long prognosis, nor given any advice on how to best care for the child. This situation contributes to the high risk for stress, isolation, depression and reduced quality of life for the caregivers. [15-18] In the absence of health professionals, training of family members has become a strategy to address the workforce gap, for example the *Caregiver Skills Training* programs for children with developmental delays and disabilities.[19] A recent initiative for CP in LMICs is a caregiver training package delivered by health workers (*Getting to know cerebral palsy; GTKCP),[20]* which aims to empower caregivers and improve care and support for children, recently studied in Ghana.[21 22] A comprehensive parent-delivered intervention program for early intervention for infants at high risk to develop CP due to adverse events at birth or shortly after, (*Learning through Everyday Activities with Parents; LEAP_CP)* is currently being evaluated in a randomized controlled trial in Bangladesh.[23] In addition to parent education, LEAP_CP comprises goal directed motor and cognitive skills training and strategies to enrich the home environment. Another early intervention parent-delivered programme, the "ABAaNA EIP" is currently being trialled in Uganda for infants under 12 months.[24] It comprises one home visit and 10 modules delivered by trained expert parents to groups of 6-10 families of infants once every one to two weeks over a six month period, There are thus several emerging intervention programmes for at-risk infants and young children with CP in low resource settings based on evidence derived from high-income countries (HICs). However, as yet, there is no evidence based programme nor a programme targeting children over two years of age in LMICs.

The neglect and lack of services and support for children with CP in LMICs is underscored by limited information about the epidemiology of CP in these countries. In 2015, our research team published the first population-based study on children with CP in sub-Saharan Africa,

For peer review only - http://bmjopen.bmj.com/site/about/guidelines.xhtml

reporting a 50% higher prevalence than HICs (a crude prevalence of 3·1/1000, [25] vs. 1·8–2·3 in HICs [26-29]). The finding of a higher prevalence of CP has been confirmed in a recent community-based key informant study from Bangladesh [30 31] and is consistent with some previous estimates from different LMIC populations. [32-35] Many children in our study had severe motor impairments often in combination with intellectual disability, impairments in communication, and seizures. None had received regular rehabilitation services. Only 8% of those unable to walk had wheelchairs, and none had assistive devices for walking, hearing, seeing or communication. Only a third of the children attended school. Notably, the Ugandan cohort of children with CP exhibited significantly lower developmental trajectories of mobility and self-care skills when compared to a similar CP cohort of Swedish children. We concluded that the needs for children with CP and their families in rural Uganda were not met, illustrated by the scarcity of health services, assistive devices, and low school attendance, and that the absence of interventions and support contributed to the poorer development of mobility and self-care skills.[4]

To address this unsettling situation, we have developed a comprehensive and holistic intervention programme for children with CP living in rural, low-resource settings, which we plan to implement and evaluate in this cohort of children with CP in rural Uganda. This Akwenda CP programme was developed by an international team of experts on CP and rehabilitation, an academic team from Uganda, a team from Malamulele Onward in South Africa and a local team of therapists and social workers living in the region in a three step process described in Supplement 1. The first step involved identifying key principles based on current concepts of disability, rehabilitation and interventions for children with CP. In the second step the team set up three general programme goals based on the principles and the needs of children with CP and their families, which had been identified in previous studies.[2 4 25 36] In the third step, the team developed the Akwenda CP programme that comprises five components, based on the principles and aimed at achieving the goals formulated in the previous steps (See Supplement 1, Supplemental Figure 1, and Supplemental Tables 1 & 2).

Aims and hypotheses

This protocol describes the method and implementation of the Akwenda CP programme study, which aims to evaluate the programme's feasibility and impact on children, caregivers and society. The specific aims are to:

1. Determine the efficacy of the intervention on the motor function, daily activities and participation of children with CP.
   Hypothesis 1: Children with CP receiving the intervention will improve their motor function, enhance their activity and increase their level of participation compared to children receiving usual care.

2. Describe the impact of the intervention on the behaviour and mental health of caregivers of children with CP.
   Hypothesis 2: Caregivers of children with CP receiving intervention will have a better

For peer review only - http://bmjopen.bmj.com/site/about/guidelines.xhtml

quality of life, reduced levels of anxiety and stress, and increased levels of knowledge and practical caregiver competence compared to caregivers receiving usual care.

3. Determine the impact of the intervention on the community awareness of childhood disability and attitudes towards children with disability and their families.
   Hypothesis 3: Stakeholders, teachers, health professionals and the lay community will have increased their knowledge about childhood disability and the barriers limiting their participation in society. Their perception of disability will become more positive.

Methods

Study design

The project is designed as a quasi-randomized controlled, clinical study conducted according to SPIRIT guidelines (see Figure 1). Children with confirmed diagnoses of CP will be assigned to either a study group or control group, based on geographic regions. The study group will receive the Akwenda CP programme during the first year, whilst the control group will be on a waitlist and receive usual care. The control group will receive the intervention after one year. Children and caregivers of the first intervention group will be encouraged to continue with the parent-led workshops and to implement new caregiving skills and behaviours during the second year. Both groups will be assessed prior to the start of the intervention, after one year and after two years.

FIGURE 1 HERE

Study site

The study will be performed at the Iganga/Mayuge Health Demographic Surveillance Site (IM-HDSS), which was set up in collaboration between Makerere University and Karolinska Institute. It comprises 65 villages including 15,964 households, and >85 000 individuals of whom approximately 50% are under 18 years. The populations are predominantly rural subsistence farmers living under the poverty level, but 20% of the population resides in the semi-urban Iganga town. The HDSS includes one general hospital (Iganga Hospital), 27 health centres and 132 private health providers.

Participants and recruitment

Participants will be from a population-based cohort of children with CP identified from previous screening carried out at IM-HDSS in 2015 using the definition of CP Surveillance in Europe.[25 37] In addition, children with CP aged 2-6 years living in the same area will be recruited by the community development officer. The CP diagnosis will be confirmed by a child neurologist using the same definitions. Hence, the study population will be between 2

For peer review only

and 23 years at the onset of the intervention study.

Randomisation and blinding

Since the community and advocacy intervention will target the community, all children from a particular village need to be in the same group. Children will thus be allocated to groups depending on the village where they reside. Neighbouring villages will be clustered together to reduce contamination between groups resulting in two geographically defined groups, which will be matched for age, sex and motor function. The decision regarding which group will receive the intervention first will be decided by a coin flip. A team of therapists and a social worker will administer the assessments and surveys before the study to capture background and baseline data. A group of independent therapists, not involved in the intervention, will administer the outcome measures after the interventions. These examiners will be blinded to group allocation.

Intervention arm

The Akwenda CP programme comprises five elements (See Figure 2; Supplement 1 and Supplemental Tables 1 and 2 for detailed description):

Caregiver-led training workshops: The core is a series of seven workshops for caregivers of children with CP. This workshop series is based on the Malamulele Onward Carer-2-Carer Training Programme (MOC2CTP).[38 39] Four caregivers of children with CP from Iganga have been identified and trained to lead the workshops following the detailed MOC2CTP-manual. (See Figure 2 and Supplemental Table 1).

Therapist-led practical group sessions and workshops: Trained paediatric occupational- and physio-therapists will run monthly practical sessions for groups of caregivers, based on the material covered in the caregiver-led workshops. The emphasis of the practical sessions is on active caregiver participation and includes training caregivers to facilitate their children's active participation and engagement. The sessions will be structured such that the therapist will be able to give each child individual attention during group activities. The parent facilitators will assist the therapists during these practical sessions. The therapists will in addition run four workshops including i) technical assistive devices; ii) goal based training; iii) nutrition and iv) epilepsy (See Figure 2 and Supplemental table 2). To ensure consistency and quality, a therapist manual was developed for the intervention.

Provision of technical assistive devices (TAD): TAD are an important part of evidence based rehabilitation, promoting greater independence and enabling children to perform tasks they were formerly unable to accomplish within mobility, self-care, eating and communication domains.[40] Each child's need for TAD will be assessed by therapists at baseline and the children and caregivers will be involved in deciding what TAD they need.

For peer review only - http://bmjopen.bmj.com/site/about/guidelines.xhtml

Examples are wheelchairs, standing frames, posture support chairs, walking devices, parallel bars, communication boards and cut out feeding cups. When possible, TAD will be locally manufactured with affordable local materials, and caregivers will be involved in cost sharing to ensure affordability and sustainability.

Goal directed training: Goal directed training is a child-active, motor learning approach. [41] Several studies have demonstrated successful goal directed programs in HICs,[42-45] while there are no reports from LMICs. The child and caregiver, supported by a therapist, will identify three realistic goals and create action plans for how to achieve them through daily training and practice in the home environment. The goal-setting procedure will start during the fourth month. By this time the therapist has become acquainted with the child, and the caregiver has attended the caregiver-led workshops and hence will have a more realistic understanding of the child's potential. Children issued TAD will have a fourth goal relating to their use.

Communication and advocacy for behavioural and social change (C&A): The C&A component will address stigma, discrimination and exclusion of children with disabilities, and is based on Communication for Development (C4D), a systematic and evidence-based strategy to promote positive social and behavioural changes.[46] The broad themes for C&A are human rights of children with disabilities and barriers to services and how to overcome them. C&A will be run by a community social worker using community meetings and radio talk shows. Community meetings will involve political and religious leaders, teachers and health workers. Caregivers and community stakeholders will participate in a joint meeting to develop a community action plan for how to make services more inclusive.

Figure 2 Here

Standard care arm

Standard or usual care refers to that available for children with CP in the study area. Currently, children have access to primary health care and the local hospital. Support and services are rare in this rural setting with no specialized services at the local public hospitals or health centres. There are some private actors and non-governmental organisations working in the surrounding area, which may provide occasional sessions of physiotherapy or occupational therapy, and unpredictable donations of technical assistive devices such as wheel chairs or crutches.[4] Caregivers in the usual care group will be asked to keep a monthly care diary during the waiting period.

Sample size

From the initial cohort of 97 children with CP, 82 children had survived.[36] In order to include younger children, we have identified 20 additional children with CP living in the area. From these two samples, 88 children have been recruited to form two comparable groups each with 44 children. In addition, the primary caregiver of each child will be included in the two arms,

For peer review only - http://bmjopen.bmj.com/site/about/guidelines.xhtml

and forty community representatives will be included in the intervention arm for evaluation of the C&A component.

To explore whether this sample size was sufficient for detecting a true effect of the intervention, we did power calculations to determine what mean difference in Pediatric Quality of Life Inventory (PEDs-QL; primary outcome measure for caregivers) scores was needed between arms. We used a PEDs-QL mean of 12.5 and standard deviation of 18.6, from a previous study in Ghana on caregivers of children with CP.[22] In our population with 44 per group allowing for a 10% non-participation at follow up, a mean difference of at least 12.5 points between groups is needed for 80% power and a type I error of 0.05. Considering that the mean improvement after a parent training intervention in Ghana in PEDS-QL was +31.3 (95%CI 25.6-37.0) this seems plausible.[22]

Measures

To evaluate the multi-dimensional study aims, a series of outcome measures will be used before (baseline) and 1 and 2 years after the intervention at the following levels: i) child, ii) caregiver, and iii) community (See Figure 3). The outcome measures were selected and modified by the international team of experts, a team from Malamulele Onward in South Africa and a local team of therapists and social workers living in the region. We selected gold standard outcome measures when possible, but since the context in LMIC differs from HIC, some tools needed to be modified, and others were selected among tools previously utilized in other low-resource settings. (See Supplement 1)

Figure 3 here

Background characteristics

Background data will be collected on all children before allocation to groups. This includes sociodemographic information and medical history; Gross Motor Function Classification System (GMFCS); Manual Ability Classification System (MACS); Communication Function Classification System (CFCS), clinical and nutritional examinations including anthropometric data; information on school attendance, care seeking, and need for and current usage of TAD.

Child related outcome measures

Gross Motor Function Measure (GMFM66): The GMFM66 will be used as the primary child related outcome. It is a clinical tool regarded as the golden standard when evaluating change in gross motor function in children with CP.[47]

Uganda version of Pediatric Evaluation of Disability Inventory (PEDI-UG): This a parent-reported measure of the child's independence in self-care, mobility and social function widely used in both research and clinical practice. The PEDI was developed in North America and has recently been culturally adapted and translated to the Ugandan context.[48 49]

For peer review only - http://bmjopen.bmj.com/site/about/guidelines.xhtml

For peer review only

Picture my Participation (PMP): PMP is an instrument designed to assess the participation of children, particularly in LMICs. [50] It measures attendance and involvement in 20 activities.

Goal attainment scale (GAS): GAS is an objective method of quantifying goal attainment that has been widely applied to children with CP. [41] Goals are scored on a 5-point, Likert-type scale from no change at all to attainment of goal at much more than the expected level. This measure will only be used in the intervention group.

Evaluation of TAD: A goal for the use of the assistive devices will be set for each child in the intervention group, and the GAS scale will be used to evaluate the use of assistive devices. User-satisfaction will be evaluated at the end of the intervention using a questionnaire based on the Quebec User Evaluation of Satisfaction with Assistive Technology adapted to Uganda.[51]

For peer review only

Caregiver related outcome measures

A Knowledge Questionnaire evaluating caregiver's learning outcomes from the caregiver-led workshops will be administered before and after the workshop. This questionnaire comprises 26 statements, with three possible responses: agree, disagree or not sure. It is based on the Knowledge Questionnaire developed to evaluate the Malamulele Onward C2CTP. It was modified during the piloting phase; two statements were removed because they were ambiguous and one statement was rewritten to provide greater clarity.

Caregivers skill and behaviours: Interaction, skills and behaviours of caregivers performing two everyday activities with their child: i.e., dressing and undressing; and feeding in the child's home will be captured in two videos, each four minutes long. The assessment of the video clips has been developed and used by Malamulele Onward and further adapted by the research team for this study. Five observable behaviours will be scored using a four point Likert scale, with 0 being "none of the time" and 3 being "90% or more of the time."

Caregivers quality of life: Caregiver quality of life will be assessed using the 36 item Pediatric Quality of Life Inventory PedsQL Family Impact Module, which is a validated and reliable measure.[52] This was successfully used in a similar study in Ghana, [21] and has been translated into *Luganda,* the local language.

Caregivers stress: Caregiver stress will be assessed using the Zimbabwe Caregiver Challenges Scale.[53] This tool was recently developed for caregivers of children with lifelong disabilities living in low-resourced African settings.

Community related outcome measures

Communication and Advocacy Survey (CAS) Questionnaire: This tool is based on the CBM (Christian Blind Mission) Inclusive Development Tool Kit [54] and comprises 44 items measuring the knowledge, attitudes, and practices of community members. The tool, available in both English and Lusoga, has been developed by the research team and pre-tested on community members in Uganda.

Data collection, management and analysis

Organization and management

The Akwenda-CP programme is a study within the CURIE research consortium, a collaboration between the Makerere University College of Health Sciences, Kampala, Uganda and Karolinska Institute, Stockholm, Sweden and clinical researchers from South Africa and USA. The project will be guided by a steering group ensuring the conduct and coordination of the study. There will be a local Site Steering Committee consisting of the PI (AKM), an administrative project manager, a PhD student, and a parent carer. The project manager will be coordinating the study on site and report to the PI.

Data collection and monitoring

Two therapists not involved in the intervention will administer the baseline and outcome assessments. They have pre-knowledge of the project from participating in the development of the intervention, and will be trained over a period of four weeks in the assessments. The PhD- student, also a therapist, will assist in the assessments and be responsible for data collection, storage and analysis. A data manager will supervise the overall organization, storage and security of the data and ensure information is recorded accurately.

Data management and analysis

Data will be collected in Open Data Kit using tablets, and electronic data will be managed through a secure database. The use of electronic data collection with built in quality control will minimize missing or inaccurately entered data. The researchers will supervise data collection and monitor the database in real-time to address any quality issues that might be arising. Data will be analysed using SPSS. Standard analysis methods for a randomised controlled trial, including general linear mixed models, will be used to make comparisons between the intervention group and the wait list group on an intention to treat basis. Effect estimate will be presented as a mean difference and 95% CI. Secondary outcome analyses will use similar methods to compare all other outcomes between groups immediately post-intervention and again one year later. The GAS goal attainment scores for the intervention group will be analysed with paired t-tests on a within-group basis. A senior statistician will guide and assist with the analysis.

Patient and public involvement statement

The Akwenda CP programme and the study design have been developed in collaboration with caregivers, community leaders and local health professionals. All of these stakeholders were invited to a dissemination event in January 2019 where the results from the first screening study from 2015 were reported. At the same time, plans for a four-year follow-up study and this intervention study were presented and discussed. Local therapists and social workers from the region, together with four caregivers, have thereafter participated in the development and adaptation of the intervention programme (See Supplement 1), and in designing and planning the study. The parent-led workshop series, a core part of the intervention programme, was developed and refined by a team of therapists and the parents of children with CP in South

For peer review only

Africa.

Ethics and dissemination

Ethics: The research project has been reviewed by the Higher Degrees Research and Ethics Committee, School of Public Health, College of Health Sciences, Makerere University (Ref: 727) and approved by the Uganda National Council for Science and Technology (UNCST) (SS 5173). Written informed consent for study participation will be obtained from the mother or legal guardian, and, if possible, assent will be obtained from children above eight years. The electronic data will be managed through a secure database. Participants' identities will not be revealed in any publications or reports emanating from this study. Video file names will be de-identified. A study protocol including all items of the WHO Trial Registration Data Set has been completed and the study has been registered in accordance with WHO and ICMJE standards at the Pan African Clinical Trials Registry (PACTR202011738099314). Any amendment of the study protocol requires approval from the Research and Ethics Committee.

Safety: As the study is not a drug trial, there is no data safety monitoring committee. A COVID-19 risk management protocol has been developed and approved by the Higher Degrees Research and Ethics Committee, School of Public Health, College of Health Sciences, Makerere University and will be strictly adhered to. Any adverse events associated with the study will be screened weekly by the treating therapists, and reported to the PI, and referred to the Ethics committee if considered serious. Any decision to describe interim results or terminate the trial will be made by the investigators with oversight from the Ethics committee.

Dissemination: Locally within Iganga-Mayuge, a stakeholders' dissemination event will be organized. Families involved in the study along with the key players in the health, community, policing, media, and disability groups will be invited to participate.

At the national level, findings will be disseminated through the Ministry of Health, Non Communicable Diseases (NCD) platform, and collaboration with health regulatory councils and professional associations will result in professional development courses that utilize the study findings to be disseminated to nearly all the health practitioners and health policy planners/implementers working in Uganda. Findings will also be disseminated through print media that will enable non-health workers to access information regarding the study findings.

For the international community, study findings will be published through Open Access, peer-reviewed scientific journals and presented at international conferences. Several authors are in the International Alliance of Academies of Childhood Disability (IAACD), and these findings will be important when developing recommendations for good clinical practice and will be disseminated through lectures and webinars organized by the IAACD.

For peer review only

DISCUSSION

To our knowledge, this is the first intervention programme for children and youth with CP in a LMIC that provides a holistic approach with a broad spectrum of interventions aimed at children and their caregivers as well as the wider community. The intervention is designed to be affordable in a low resource setting where many families live at or below the poverty level, and with a scarcity of therapists and other rehabilitation professionals. The core of the intervention is a carer-to-carer training programme. Intervention programmes in Ghana and West Bengal have previously used parent training programmes, [21 23] however, that training was either delivered by health workers or community disability workers, only some of whom were mothers of children with disabilities.

Currently, few evidence-based interventions have been evaluated in LMICs. The activities of the Akwenda CP programme are based on best clinical practice derived from clinical studies in HICs, and adapted to the conditions in this rural part of Uganda in accordance with the WHO's goal of universal health coverage and universal access.[55] The multiple activities add to the complexity of the intervention as well as the evaluation. It will therefore not be possible to study the effects of individual components. Our aim is rather to study the impact of the entire intervention as well as the programme's feasibility, cost implications, effectiveness, accessibility and acceptability.

Most intervention studies focus on children under the age of 12 years.[21-24] Our study includes a much wider age range, up to the age of 23 years. The majority of children and youth have not had access to early intervention or to regular therapy, which is the situation in many LMICs. By including a wider age range of participants, findings from this study will be relevant to all age groups, not just younger children. Information from this study will also shed light on the extent to which positive change is still possible in youth with CP who have received very little intervention during early life.

Author contributions:

GS was leading the development of the intervention programme based on her previous experience at Malamulele Onward. She was also involved in designing the study protocol, drafted the manuscript and approved the final manuscript as submitted.

FE was involved in leading the intervention programme development, in particular the C&A component, and taking part in the design of the study protocol and drafted the manuscript and approved the final manuscript as submitted.

CA has been involved in programme development and design of the study protocol and has revised drafts of the manuscript and approved the final manuscript as submitted.

DD has been involved in programme development and design of the study protocol and has revised drafts of the manuscript and approved the final manuscript as submitted.

AKM conceptualised the study and was involved in programme development and design of the study protocol and revised drafts of the manuscript and approved the final manuscript as submitted.

For peer review only - http://bmjopen.bmj.com/site/about/guidelines.xhtml

HF conceptualised the study, secured funding for the study, was involved in programme development and led the design of the study protocol and drafted the manuscript and approved the final manuscript and was responsible for submission.

Collaborators:

The development of the intervention was done in collaboration with a team of four local therapists from the region; Sauba Kamusiime OT, Catherine Ntanya OT, Edward Opio OT, and Eric Kintu PT, one social worker; Godfrey Wanjala from Iganga, four caregivers; Babra Esaete, Margret Nalweiso, Rose Naigaga, and Robinah Nakaziba who were trained to become Parent Facilitators by Lydia Ngwana, a master trainer from Malamulele Onward in Johannesburg, South Africa. The Parent Facilitators and master trainer were also involved in adapting the carer-to-carer programme to the local conditions in rural Uganda. Keron Ssebyala was involved in the project administration, coordination, and also supported the practical work in planning the intervention programme and study, and the training of the staff. Tom Babalanda was involved in coordinating logistical requirements during field activities including training. IM-HDSS supported with data management within their system at the Iganga field site.

Funding: We acknowledge the funding of the project by the Swedish Research Council (2017-05474) and by the Foundation Frimurare Barnhuset in Stockholm. The funders had no role in study design; collection, management, analysis, and interpretation of data; writing of the report; and the decision to submit the report for publication.

Competing interests: Non declared

Patient consent: Will be required for children and families participating in the study.

Data Sharing Statement: The clinical protocol has been registered in accordance with WHO and ICMJE standards at the Pan African Clinical Trials Registry (PACTR202011738099314).The data collected in the clinical trial used in the scientific reports will be uploaded at the Swedish National Data Service repository and shared without restrictions: SND-ID: 2020-181; Reserved DOI: 10.5878/97y5-5h14; https://snd.gu.se/en/catalogue/study/preview/4e2353f2-e277-42a1-8f0d-5281b3f22153.

Open access: This is an open access article distributed in accordance with the Creative Commons Attribution 4.0 Unported (CC BY 4.0) license, which permits others to copy, redistribute, remix, transform and build upon this work for any purpose, provided the original work is properly cited, a link to the licence is given, and indication of whether changes were made. See: https:// creativecommons. org/ licenses/ by/ 4. 0/.

Figure 1: Flow-chart for children in the Akwenda Cerebral Palsy Intervention Study

Figure 2: Timetable for delivering the intervention through the five components over a period of 11 months.

For peer review only - http://bmjopen.bmj.com/site/about/guidelines.xhtml

Figure 3: Akwenda-CP programme overview with the five components, three levels, three goals and all outcome measures. See Supplement for detailed background and design of the programme.

REFERENCES

1. Rosenbaum P, Paneth N, Leviton A, et al. A report: the definition and classification of cerebral palsy April 2006. *Dev Med Child Neurol Suppl* 2007;109:8-14. [published Online First: 2007/03/21]

2. Kakooza-Mwesige A, Tumwine JK, Eliasson AC, et al. Malnutrition is common in Ugandan children with cerebral palsy, particularly those over the age of five and those who had neonatal complications. *Acta Paediatr* 2015;104(12):1259-68. doi: 10.1111/apa.13089 [published Online First: 2015/06/20]

3. Kuper H, Monteath-van Dok A, Wing K, et al. The impact of disability on the lives of children; cross-sectional data including 8,900 children with disabilities and 898,834 children without disabilities across 30 countries. *PLoS One* 2014;9(9):e107300. doi: 10.1371/journal.pone.0107300

4. Andrews C, Kakooza-Mwesige A, Almeida R, et al. Impairments, functional limitations, and access to services and education for children with cerebral palsy in Uganda: a population-based study. *Dev Med Child Neurol* 2020;62(4):454-62. doi: 10.1111/dmcn.14401

5. Bunning K, Gona JK, Newton CR, et al. The perception of disability by community groups: Stories of local understanding, beliefs and challenges in a rural part of Kenya. *PLoS One* 2017;12(8):e0182214. doi: 10.1371/journal.pone.0182214

6. Kane JC, Elafros MA, Murray SM, et al. A scoping review of health-related stigma outcomes for high-burden diseases in low- and middle-income countries. *BMC Med* 2019;17(1):17. doi: 10.1186/s12916-019-1250-8

7. Mitter N, Ali A, Scior K. Stigma experienced by families of individuals with intellectual disabilities and autism: A systematic review. *Res Dev Disabil* 2019;89:10-21. doi: 10.1016/j.ridd.2019.03.001

8. Bright T, Wallace S, Kuper H. A Systematic Review of Access to Rehabilitation for People with Disabilities in Low- and Middle-Income Countries. *Int J Environ Res Public Health* 2018;15(10) doi: 10.3390/ijerph15102165

9. Gupta N, Castillo-Laborde C, Landry MD. Health-related rehabilitation services: assessing the global supply of and need for human resources. *BMC Health Serv Res* 2011;11:276. doi: 10.1186/1472-6963-11-276

10. World_Health_Organization. Rehabilitation 2030: A call for action 2017 [Available from: http://www.

who.int/disabilities/care/rehab-2030/en/

11. Matter R, Harniss M, Oderud T, et al. Assistive technology in resource-limited environments: a scoping review. *Disabil Rehabil Assist Technol* 2017;12(2):105-14. doi: 10.1080/17483107.2016.1188170

For peer review only - http://bmjopen.bmj.com/site/about/guidelines.xhtml

12. Donald KA, Kakooza AM, Wammanda RD, et al. Pediatric Cerebral Palsy in Africa: Where Are We? *J Child Neurol* 2015;30(8):963-71. doi: 10.1177/0883073814549245 [published Online First: 2014/10/10]

13. Wilmshurst JM, Badoe E, Wammanda RD, et al. Child neurology services in Africa. *J Child Neurol* 2011;26(12):1555-63. doi: 10.1177/0883073811420601

14. Scherzer AL, Chhagan M, Kauchali S, et al. Global perspective on early diagnosis and intervention for children with developmental delays and disabilities. *Dev Med Child Neurol* 2012;54(12):1079-84. doi: 10.1111/j.1469-8749.2012.04348.x

15. Gona JK, Mung'ala-Odera V, Newton CR, et al. Caring for children with disabilities in Kilifi, Kenya: what is the carer's experience? *Child Care Health Dev* 2011;37(2):175-83. doi: 10.1111/j.1365-2214.2010.01124.x [published Online First: 2010/07/22]

16. Hartley S, Ojwang P, Baguwemu A, et al. How do carers of disabled children cope? The Ugandan perspective. *Child Care Health Dev* 2005;31(2):167-80. doi: 10.1111/j.1365-2214.2004.00464.x

17. Mobarak R, Khan NZ, Munir S, et al. Predictors of stress in mothers of children with cerebral palsy in Bangladesh. *J Pediatr Psychol* 2000;25(6):427-33. doi: 10.1093/jpepsy/25.6.427

18. Dambi JM, Jelsma J, Mlambo T. Caring for a child with Cerebral Palsy: The experience of Zimbabwean mothers. *Afr J Disabil* 2015;4(1):168. doi: 10.4102/ajod.v4i1.168

19. WHO. Training parents to transform children's lives 2020 [Available from: https://www.who.int/mental_health/maternal-child/PST/en/.

20. GTKCP. Working with parent groups – a training resource for facilitators, parents, caregivers, and persons with cerebral palsy 2020 [Available from: https://www.lshtm.ac.uk/sites/default/files/2019-06/Getting-to-know-cerebral-palsy-english.pdf.

21. Zuurmond M, Nyante G, Baltussen M, et al. A support programme for caregivers of children with disabilities in Ghana: Understanding the impact on the wellbeing of caregivers. *Child Care Health Dev* 2019;45(1):45-53. doi: 10.1111/cch.12618

22. Zuurmond M, O'Banion D, Gladstone M, et al. Evaluating the impact of a community-based parent training programme for children with cerebral palsy in Ghana. *PLoS One* 2018;13(9):e0202096. doi: 10.1371/journal.pone.0202096

23. Benfer KA, Novak I, Morgan C, et al. Community-based parent-delivered early detection and intervention programme for infants at high risk of cerebral palsy in a low-resource country (Learning through Everyday Activities with Parents (LEAP-CP): protocol for a randomised controlled trial. *BMJ Open* 2018;8(6):e021186. doi: 10.1136/bmjopen-2017-021186

24. Nampijja M, Webb E, Nanyunja C, et al. Randomised controlled pilot feasibility trial of an early intervention programme for young infants with neurodevelopmental impairment in Uganda: a study protocol. *BMJ Open* 2019;9(10):e032705. doi: 10.1136/bmjopen-2019-032705

25. Kakooza-Mwesige A, Andrews C, Peterson S, et al. Prevalence of cerebral palsy in Uganda: a population-based study. *The Lancet Global health* 2017;5(12):e1275-e82. doi: 10.1016/s2214-109x(17)30374-1 [published Online First: 2017/11/06]

26. Oskoui M, Coutinho F, Dykeman J, et al. An update on the prevalence of cerebral palsy: a systematic review and meta-analysis. *Developmental Medicine and Child Neurology* 2013;55(6):509-19. doi: 10.1111/dmcn.12080

For peer review only - http://bmjopen.bmj.com/site/about/guidelines.xhtml

27. Sellier E, Platt MJ, Andersen GL, et al. Decreasing prevalence in cerebral palsy: a multi-site European population-based study, 1980 to 2003. *Dev Med Child Neurol* 2016;58(1):85-92. doi: 10.1111/dmcn.12865

28. Van Naarden Braun K, Doernberg N, Schieve L, et al. Birth Prevalence of Cerebral Palsy: A Population-Based Study. *Pediatrics* 2016;137(1) doi: 10.1542/peds.2015-2872 [published Online First: 2015/12/15]

29. Smithers-Sheedy H, McIntyre S, Gibson C, et al. A special supplement: findings from the Australian Cerebral Palsy Register, birth years 1993 to 2006. *Dev Med Child Neurol* 2016;58 Suppl 2:5-10. doi: 10.1111/dmcn.13026

30. Khandaker G, Muhit M, Karim T, et al. Epidemiology of cerebral palsy in Bangladesh: a population-based surveillance study. *Dev Med Child Neurol* 2018 doi: 10.1111/dmcn.14013

31. Andrews C, Kakooza-Mwesige A, Eliasson AC, et al. Important report on cerebral palsy in Bangladesh: but different findings compared with other countries need further exploration. *Dev Med Child Neurol* 2019 doi: 10.1111/dmcn.14202

32. El-Tallawy HN, Farghaly WM, Shehata GA, et al. Cerebral palsy in Al-Quseir City, Egypt: prevalence, subtypes, and risk factors. *Neuropsychiatr Dis Treat* 2014;10:1267-72. doi: 10.2147/NDT.S59599

33. Chang MJ, Ma HI, Lu TH. Estimating the prevalence of cerebral palsy in Taiwan: A comparison of different case definitions. *Res Dev Disabil* 2014;36C:207-12. doi: 10.1016/j.ridd.2014.10.001

34. Banerjee TK, Hazra A, Biswas A, et al. Neurological disorders in children and adolescents. *Indian J Pediatr* 2009;76(2):139-46. doi: 10.1007/s12098-008-0226-z

35. Couper J. Prevalence of childhood disability in rural KwaZulu-Natal. *S Afr Med J* 2002;92(7):549-52.

36. Namaganda LH, Almeida R, Kajungu D, et al. Excessive premature mortality among children with cerebral palsy in rural Uganda: A longitudinal, population-based study. *PLoS One* 2020;15(12):e0243948. doi: 10.1371/journal.pone.0243948

37. Surveillance of Cerebral Palsy in E. Surveillance of cerebral palsy in Europe: a collaboration of cerebral palsy surveys and registers. Surveillance of Cerebral Palsy in Europe (SCPE). *Dev Med Child Neurol* 2000;42(12):816-24.

38. Onward M. CARER-2-CARER TRAINING PROGRAMME 2020 [Available from: https://www.malamuleleonward.org/what-we-offer/carer-2-carer-programme.

39. Saloojee G, Bezuidenhout M. Parent-led services and community-based peer supporters for children and adults with disabilities: experiences from two programmes. In: Padarath A, Kathard H, Lorenzo T, et al., eds. South African Health Review 2020. Durban: Health Systems Trust 2020.

40. WHO. Global Cooperation on Assistive Technology (GATE) 2020 [Available from: https://www.who.int/disabilities/technology/gate/en/.

41. Ketelaar M, Vermeer A, Hart H, et al. Effects of a functional therapy program on motor abilities of children with cerebral palsy. *Phys Ther* 2001;81(9):1534-45. doi: 10.1093/ptj/81.9.1534

42. Ahl LE, Johansson E, Granat T, et al. Functional therapy for children with cerebral palsy: an ecological approach. *Dev Med Child Neurol* 2005;47(9):613-9.

43. Bower E, McLellan DL, Arney J, et al. A randomised controlled trial of different intensities of physiotherapy and different goal-setting procedures in 44 children with cerebral palsy. *Dev Med Child Neurol* 1996;38(3):226-37. doi: 10.1111/j.1469-8749.1996.tb15084.x

For peer review only

44. Lowing K, Bexelius A, Brogren Carlberg E. Activity focused and goal directed therapy for children with cerebral palsy--do goals make a difference? *Disabil Rehabil* 2009;31(22):1808-16. doi: 10.1080/09638280902822278

45. Lowing K, Bexelius A, Carlberg EB. Goal-directed functional therapy: a longitudinal study on gross motor function in children with cerebral palsy. *Disabil Rehabil* 2010;32(11):908-16. doi: 10.3109/09638280903353422

46. UNICEF. UNICEF 2017 Report on Communication for Development (C4D) 2017 [Available from: https://www.unicef.org/media/47781/file/UNICEF_2017_Report_on_Communication_for_Development_C4D.pdf.

47. Russell DJ, Avery LM, Rosenbaum PL, et al. Improved scaling of the gross motor function measure for children with cerebral palsy: evidence of reliability and validity. *Phys Ther* 2000;80(9):873-85. [published Online First: 2000/08/29]

48. Kakooza-Mwesige A, Tumwine JK, Forssberg H, et al. The Uganda version of the Pediatric Evaluation of Disability Inventory (PEDI). Part I: Cross-cultural adaptation. *Child Care Health Dev* 2018;44(4):552-61. doi: 10.1111/cch.12563 [published Online First: 2018/03/13]

49. Amer A, Kakooza-Mwesige A, Jarl G, et al. The Ugandan version of the Pediatric Evaluation of Disability Inventory (PEDI-UG). Part II: Psychometric properties. *Child Care Health Dev* 2018;44(4):562-71. doi: 10.1111/cch.12562

50. Arvidsson P, Dada S, Granlund M, et al. Content validity and usefulness of Picture My Participation for measuring participation in children with and without intellectual disability in South Africa and Sweden. *Scand J Occup Ther* 2019:1-13. doi: 10.1080/11038128.2019.1645878

51. Demers L, Weiss-Lambrou R, Ska B. Development of the Quebec User Evaluation of Satisfaction with assistive Technology (QUEST). *Assist Technol* 1996;8(1):3-13. doi: 10.1080/10400435.1996.10132268 [published Online First: 1995/12/09]

52. Varni JW, Seid M, Kurtin PS. PedsQL 4.0: reliability and validity of the Pediatric Quality of Life Inventory version 4.0 generic core scales in healthy and patient populations. *Med Care* 2001;39(8):800-12.

53. Dambi JM, Tapera L, Chiwaridzo M, et al. Psychometric evaluation of the Shona version of the Multidimensional Scale of Perceived Social Support Scale (MSPSS-Shona) in adult informal caregivers of patients with cancer in Harare, Zimbabwe. *Malawi Med J* 2017;29(2):89-96. doi: 10.4314/mmj.v29i2.3

54. CBM. Disability Inclusive Development Toolkit 2017 [Available from: https://www.cbm.org/fileadmin/user_upload/Publications/CBM-DID-TOOLKIT-accessible.pdf.

55. Evans DB, Hsu J, Boerma T. Universal health coverage and universal access: World Health Organization; 2013 [Available from: http://dx.doi.org/10.2471/BLT.13.125450 [PDF].

For peer review only

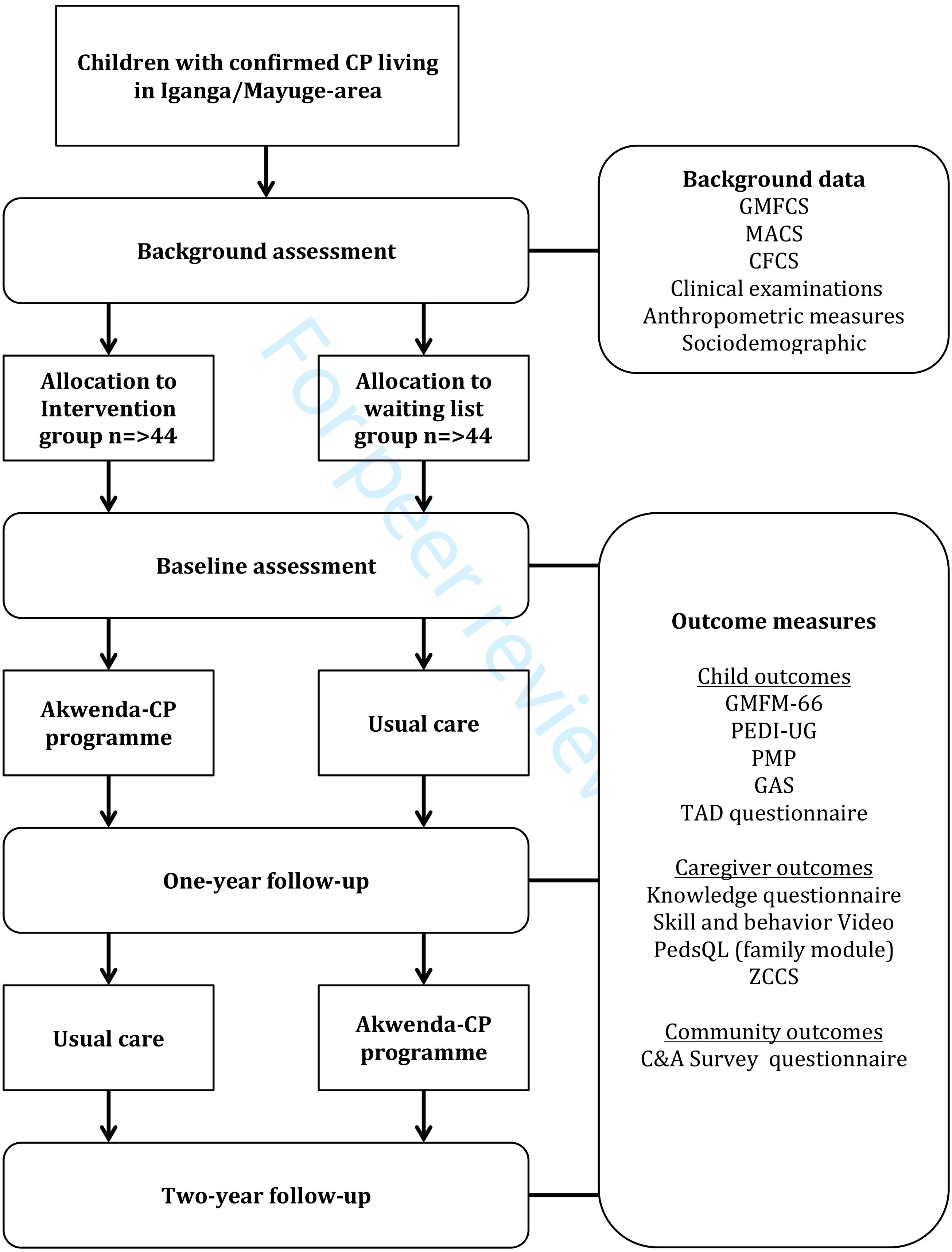
Children with confirmed CP living in Iganga/Mayuge-area
Background assessment
Background data
GMFCS
MACS
CFCS
Clinical examinations
Anthropometric measures
Sociodemographic
Allocation to Intervention group n=>44
Allocation to waiting list group n=>44
Baseline assessment
Akwenda-CP programme
Usual care
One-year follow-up
Usual care
Akwenda-CP programme
Two-year follow-up
Outcome measures
Child outcomes
GMFM-66
PEDI-UG
PMP
GAS
TAD questionnaire
Caregiver outcomes
Knowledge questionnaire
Skill and behavior Video
PedsQL (family module)
ZCCS
Community outcomes
C&A Survey questionnaire

Components	Activities over the 11 months of the Akwenda CP intervention programme										
	1	2	3	4	5	6	7	8	9	10	11
**Caregiver-led training workshops**	What is CP?	CP as a way of life			Getting my child's body ready to be active	Eating & drinking	Communi-cation		Play	CVI	Recap
**Therapist-led practical group sessions and workshops**	What is CP?	CP as a way of life	Purpose use and maintenance of TAD	Setting realistic goals for my child	Getting my child's body ready to be active	Eating & drinking	Communi-cation	Nutrition & epilepsy	Play	CVI	Recap
**Goal setting**				Group workshop: refining the goals for my child	Home visit: Confirm goals & make individual action plan			Home visit: follow up goals & TAD			Review goals (GAS)
**Technical assistive devices (TAD)**	Decide on TAD for each child			Home visit: give out and fit TAD							
**Communication & Advocacy caregivers**	Rights of children with disabilities	Mapping of available services			Barriers to services for children with CP	Barriers to services for children with CP	Joint dialogue meeting: overcoming barriers and developing a community action plan				
**Communication & Advocacy stakeholders**		What is CP? Barriers to inclusion							Follow up of community action plan by community social worker		

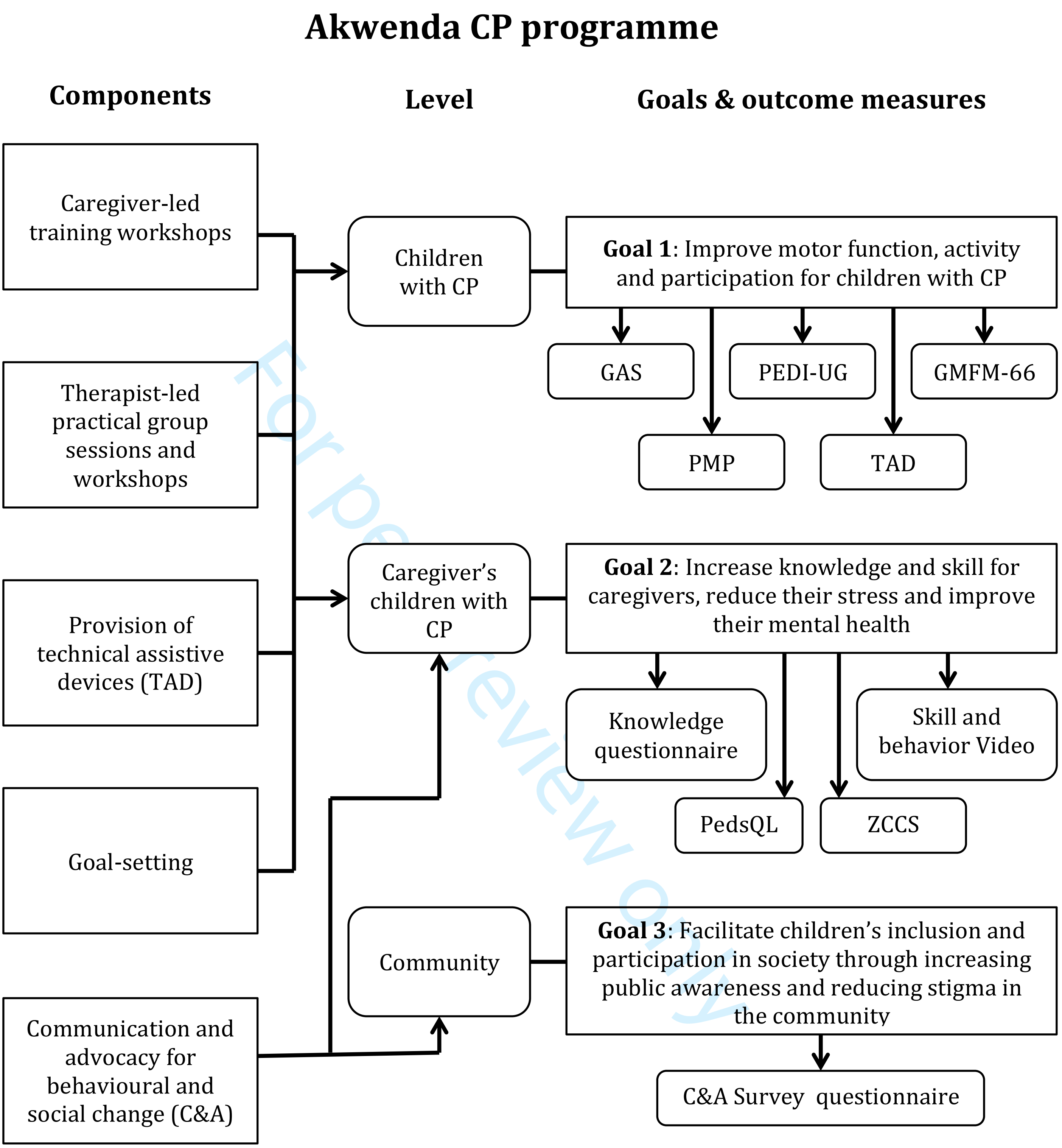
Akwenda CP programme
Components
Level
Goals & outcome measures
Caregiver-led training workshops
Therapist-led practical group sessions and workshops
Provision of technical assistive devices (TAD)
Goal-setting
Communication and advocacy for behavioural and social change (C&A)
Children with CP
Caregiver's children with CP
Community
Goal 1: Improve motor function, activity and participation for children with CP
GAS
PEDI-UG
GMFM-66
PMP
TAD
Goal 2: Increase knowledge and skill for caregivers, reduce their stress and improve their mental health
Knowledge questionnaire
Skill and behavior Video
PedsQL
ZCCS
Goal 3: Facilitate children's inclusion and participation in society through increasing public awareness and reducing stigma in the community
C&A Survey questionnaire

Supplement 1:

The Akwenda cerebral palsy intervention programme

The Akwenda cerebral palsy (CP) programme was developed by an international team of experts within childhood disabilities and rehabilitation, an academic team from Uganda, a team from Malamulele Onward in South Africa, a team of therapists and a social worker working in Eastern Uganda, as well as four caregivers from the study area. Findings from our previous studies in the same cohort identifying the needs among the children and families, and the gaps in service provision also informed the process. The first step identified key principles based on current concepts of disability, rehabilitation and interventions for children with CP which would be possible to implement in low-resource settings. This involved reviewing literature and learning from programmes developed for low-resource settings with no or few health professionals. From this review,, the parent-led Carer-2-Carer Training Programme developed and implemented by Malamulele Onward in Johannesburg, South Africa was chosen to form the core of the Akwenda programme.[1] The Malamulele Onward Carer-2-Carer programme was chosen because it targeted children with CP over the age of two years; it had been successfully used in Southern African settings for a number of years; and it was specifically designed to be delivered by parents with low literacy levels.[2] The second step involved clarifying the purpose of and agreeing on goals for the intervention programme. Based on the principles and goals formulated in previous steps, the five components of the intervention were defined in the third step.

Step 1: Key principles

The ICF framework

The first principle is the International Classification of Functioning and Disability and Health (ICF) framework which defines disability not as a characteristic of a person but as the interaction between health conditions and environmental and personal factors at the level of: i) body structure and function; ii) activity, and iii) participation in the family and society. The ICF has had an immense influence on changing perspectives of disability over the last decades in shifting the primary focus of interventions from impairments to participation and inclusion whilst emphasizing the need to address barriers, e.g., attitudinal, institutional and environmental.[3] The ICF framework has enabled community-based services to be brought closer to the children. It has also facilitated family oriented services, i.e. services that directly target the family's needs for information, education and psychosocial support.[4] The Akwenda CP programme has adopted the ICFs holistic view on disability whereby activity and participation are the main focuses of the programme. The programme also embraces the

For peer review only

community-based and family oriented concept, through utilizing trained caregivers to train other caregivers, and by moving the services into the communities and homes of the participants.

Evidence based best practice

The second principle was to ensure that the Akwenda CP programme was based on evidence based best practice. A plethora of interventions are used today for children with CP, many without supportive scientific data.[5] The past decades have seen a surge of clinical trials resulting in a wider range of evidence based interventions, as well as interventions with strong evidence that they are not effective.[5] This emerging evidence shows that interventions improving functional ability in children with CP are child-active approaches where the child is actively practicing real-life tasks. Such child-active approaches are supported by current neuroscience demonstrating activity dependent learning induced plasticity in the brain underlying the learning and improvement of motor skills.[6] With little available information on evidence-based practices from LMIC, the activities of the Akwenda programme are therefore based on best clinical practice derived from clinical studies in HIC, adapted to the low income context in this rural part of Uganda. We have in addition also sought inspiration from programmes and studies done in LMIC which provide innovative strategies to provide the best care available given limited resources. [7 8]

Universal health coverage and universal access

The third principle is based on the WHO's goal of universal health coverage and universal access, which aims to ensure that all people obtain the health and rehabilitation services they need without fear of financial hardship.[9] To achieve universal health and rehabilitation coverage, services should be physically accessible, affordable and acceptable to patients. This means that services need to be available; located close to people; and should not entail financial hardship. Not only the expense of the health services but also the indirect and opportunity costs, e.g. transportation and time away from work need to be taken into account. Acceptability captures people's willingness to seek services, and depends on how patients perceive services and how social and cultural attitudes of the health provider encourage or discourage them from seeking services.[9] We have tried to ensure that the Akwenda CP programme is physically accessible, affordable and acceptable. Physical accessibility and affordability will be ensured through locating the activities in the community, utilising locally available resources for technical assistive devices, optimising the limited therapist time, and the utilization of trained caregivers. The programme also includes a communication and advocacy component aimed to ensure acceptability.

Step 2: Goals

Three general goals based on the principles and the needs of children and youth with cerebral palsy and their families, which had been identified in previous studies[10-12] were agreed upon:

- To improve motor function, activity and participation of the children and youth with cerebral palsy
- To increase knowledge and skill of the caregivers, reduce their stress and improve their mental health

- To facilitate children's inclusion and participation in society through increasing public awareness and reducing stigma in the community.

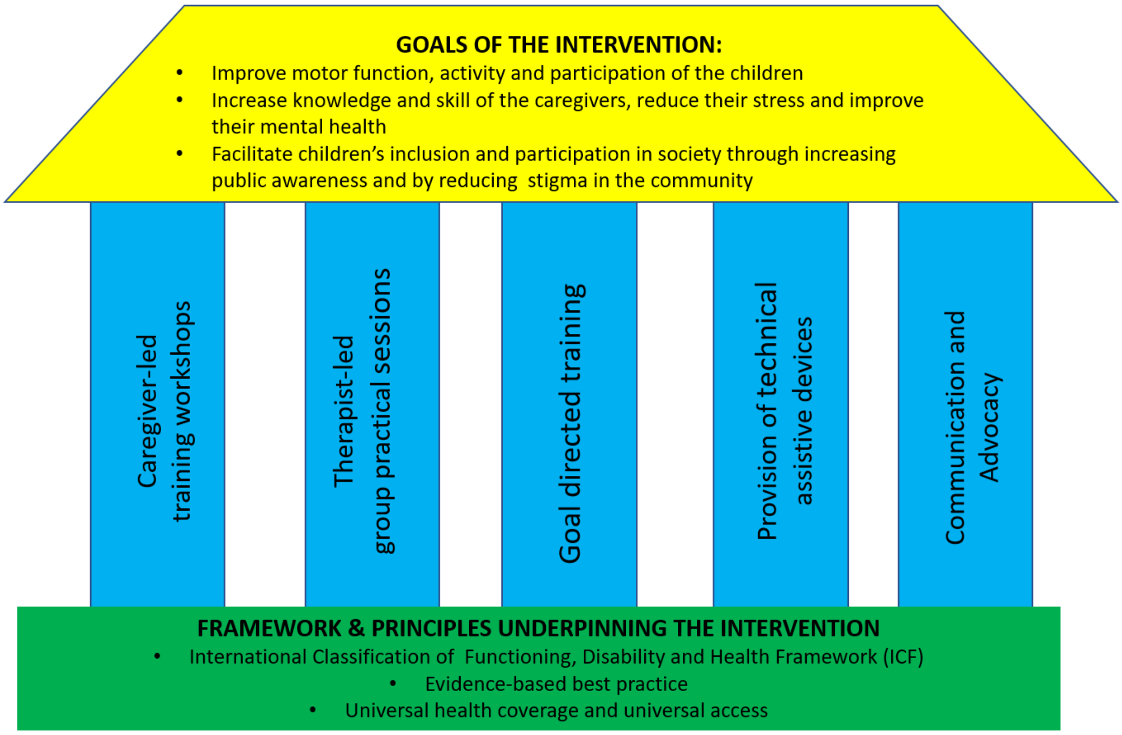

Figure 1: Illustration of the Akwenda CP programme: based on three key principles; comprising five intervention components; and aiming to achieve three goals.

Step 3: Five intervention components

Based on the principles and goals formulated in previous steps, the five components of the Akwenda CP programme are (See Figure1):

1. Caregiver-led training workshops
2. Therapist-led practical group sessions and workshops
3. Provision of technical assistive devices (TAD)
4. Goal directed trainng
5. Communication and advocacy for behavioural and social change (C&A)

Component 1: Caregiver-led training workshops

The caregiver-led training workshops of the Malamulele Onward Carer-2-Carer Training Programme (MOC2CTP) form the cores of the Akwenda CP programme.[1 2] The MOC2CTP trains primary caregivers of children with CP to be Parent Facilitators, able to organise and lead seven training workshops (Table 1). The MOC2CTP was developed and refined over a seven-year period by a multidisciplinary team of three therapists. It differs from the ABAaNA

parent-led early intervention programme in that it (i) was intentionally designed to be run by parents with low literacy levels, hence it uses very simple language and a simple layout with step-by-step instructions; (ii) was designed to be run by parents independent of a therapist being present; (iii) uses a problem solving approach based on functional classification and the main clinical subtypes and (iv) the target group is children over the age of two years. The original group of 20 Parent Facilitators themselves were major contributors to the final product through their feedback and experiences in running the programme.

	**Title of workshop**	**Outcomes**
1	What is CP and how does it affect my child?	• Be able to explain what CP is to a family member<br>• Know the GMFCS Level the child falls into<br>• Know what kind (subtype) of CP the child has<br>• Understand the possible causes of CP, and what does not cause CP, especially that CP is not a disease and not her fault
2	CP as a way of life	• What does "Caring for child with CP is a way of life" mean<br>• How children with CP change over time<br>• How we can do everyday things in a way that is helpful to children with CP<br>• Helpful ways to hold and move children with CP<br>• Helpful ways to put children with CP in different positions
3	Getting my child's body ready to move and be active	• How to get a child's body ready to move and play<br>• How to position a child in a helpful way<br>• How to use these ideas in everyday activities
4	Eating and drinking: making mealtimes safe and comfortable for my child	• How to best position a child with CP for feeding<br>• Why it is important to feed a child with CP slowly<br>• How to prepare the child's lips and mouth for eating<br>• How to help a child with CP to eat better with a spoon<br>• How to help a child with CP to learn to chew<br>• How to help a child feed himself<br>• How to help a child with CP learn to drink from a cup<br>• How to help children who drool
5	Communication: my child and I understanding each other	• What we mean by communication<br>• The different ways we can communicate<br>• How to encourage a child with CP to communicate<br>• How to help a child with CP to understand better<br>• How to help a child with CP to talk better<br>• Helping children to use signs
6	Play: unlocking my child's potential	• What is play and why it is important for a child with CP<br>• How children usually play<br>• Why it is difficult for children with CP to play<br>• How we can make it easier for all children with CP to play<br>• What we can play with
7	CVI: Understanding where and what my child can see	• Understand vision difficulties<br>• What does CVI stand for and what does it mean<br>• How to know if a child has a CVI<br>• How to play with a child with a CVI<br>• How we use what the child sees best in everyday life

Table 1: Parent led workshops based on Malamulele Onward Carer-2-Carer Training Programme (each workshop runs for 2 – 2 ½ hours).

For peer review only - http://bmjopen.bmj.com/site/about/guidelines.xhtml

To implement the Akwenda CP programme, four caregivers (three mothers and one aunt) from Iganga were identified based on their availability, motivation, initiative, interest, and communication skills. They received four weeks training from a master trainer from Malamulele Onward, herself a parent of a child with CP.

Each of the seven trainings is approximately two hours long and will be led by two Iganga Parent Facilitators working as a team. To ensure quality and consistency of the workshops, the Parent Facilitators will follow the detailed Malamulele Onward Carer-2-Carer Training Programme manual which has been translated into Lusoga, the local language. For each workshop the Parent Facilitators have a workshop kit, which includes display materials. No therapist will be involved in these seven caregiver-led workshops.

Component 2: Therapist-led practical group sessions and workshops

Two weeks following each caregiver-led workshop, caregivers and children will attend a therapist-led practical group sessions that will have the same themes as the caregiver-led training workshop (Table 1). The therapists will be assisted by the Parent Facilitators during these practical sessions. Active parent participation is an essential feature of the group practical sessions which will be structured in such a way that the therapist will be able to give each child individual attention during the group activities.

In addition, the therapists involved in the intervention will run four group training workshops on: i) technical assistive devices; ii) goal directed training and setting realistic goals; iii) nutrition and iv) epilepsy (Table 2). In the previous study on this cohort of children with CP we noted that almost half (46%) of the children were severely malnourished, and 35% of the children suffered from seizures.[13] A manual for these sessions have been developed to ensure quality and consistency.

	Title of workshop	Outcomes
1	Healthy diet and healthy eating	• Know the seven major classes of nutrients<br>• Know how to prepare a meal from locally available nutrient dense foods<br>• Understand responsive feeding practices (e.g. frequency of feeding; creating a safe and clean environment for feeding)
2	Understanding epilepsy	• How to recognise a seizure<br>• Understand misconceptions related to epilepsy and how to deal with stigma<br>• Know when and where to seek care for a child with seizures<br>• Understand the role of traditional healers and traditional treatments<br>• Understand anti-epileptic medicine – how to administer; side effects and the importance of adherence

Table 2: Therapist-led training workshops on nutrition and seizures (each workshop is one hour long)

For peer review only

The two therapists running these practical sessions are experienced in working with children with CP in rural areas of Uganda, and have received 12 days of training on the practical management of children with CP. This training included essential concepts and components of the intervention programme viz., functional activities; the child's active participation and the value of self-generated movements; goal setting; working in groups; working in partnership with parents; technical assistive devices; nutrition and epilepsy. A therapist manual detailing the structure and content of each practical session has been drawn up for the intervention.

Component 3: Provision of technical assistive devices (TAD)

Provision of technical assistive devices (TAD) is an important part of evidence based best practice as they promote greater independence and enables children to perform tasks they were formerly unable to accomplish within the domains of mobility, self-care, eating and communication.[14] In this study, the aim of provision of TAD will be to simplify caregiving and increase the child's activity and participation.

The therapists will together with the caregivers and children assess the needs of TAD during the baseline assessments. Children and youth with lower limb contractures who would benefit from the provision of a standing frame or walking device will be referred to the local hospital for serial casting if it is thought that serial casting will provide sufficient soft tissue length to enable the child or youth to stand in alignment and with their feet in an acceptable position.

Where possible, TAD will be locally manufactured with affordable local materials, and the caregiver will be involved in cost sharing to ensure affordability and sustainability. Postural mobility devices, e.g., wheelchairs or buggies are costly and difficult to make locally, and will be procured from the Ministry of Health's accredited assistive devices production unit and/or donor agencies.

Children at GMFCS III-IV will be provided with standing frames, customised posture support chairs, walking assistive device and parallel bars f all of which will be made for use in the home environment by local artisans from either wood or using appropriate paper technology. Simple communication books or boards will be provided for children with communication difficulties. Children and youth will be issued with re-usable nappies or sanitary pads if needed. Other devices will be procured or created by the treating therapist in collaboration with the primary caregiver as the need arises, such as the construction of an accessible toilet seat, cut out feeding cups, shoes, splints, and appropriate toys.

The TAD will be issued during the 3rd month of the intervention following a therapist-led training workshop explaining the use, maintenance and care of assistive devices. The therapist together with the community social worker will deliver the TAD to the child's home where they will also ensure correct fitting of the device. Usage of the TAD will be evaluated during a home visit in the 8th month.

For peer review only

Component 4: Goal directed training

Goal directed training is a child-active, motor learning approach, and includes daily practice directly targeting the achievement of a goal.[15] The child and family, supported by health workers, identify realistic goals and create action plans for how to achieve them. Several studies have demonstrated the success of goal directed programs in HIC,[16-19] while there are no reports from LMIC. The goal-setting procedure in the Akwenda CP programme will start during the fourth month when the therapist has studied the assessment results of the child and become acquainted with the child and caregiver and the caregiver has attended the first two caregiver-led training workshops, meaning that she/he has a better understanding of the child's condition and potential. The therapist will lead two consecutive group sessions to explain the concept of goal setting to parents, during which the parents will assist one another in identifying goal-areas for their child. Following the group sessions, the therapists will visit each child in their home setting where the child and caregiver supported by the therapist will set three goals based on the goal-areas identified by the parents. An individualised activity plan will thereafter be made for each child, with the aim of achieving the goals within five months through daily training and practice in the home environment. Assessments performed before the intervention begins, including the Gross Motor Function Measurement (GMFM),[20] the Ugandan version of the Pediatric Evaluation of Disability Inventory (PEDI-UG), [21 22] and Picture my Participation measure,[23] will be used to inform the goal-setting procedure by identifying realistic goals based on the child's abilities and potential. These assessments explore different dimensions within the ICF framework, i.e., functional limitations, ability to perform activities and participation.

Each of the three goals will be scaled by the therapist as goal attainment scale (GAS) goals. For children issued with TAD, a fourth GAS goal will relate to the use of these TAD. Three months later, all GAS goals and the individualised activity plans for each child will be reviewed during the monthly group session or at a home visit; and re-evaluated in the 11th month.

Component 5: Communication and Advocacy for behavioural and social change (C&A)

This component is based on Communication for Development (C4D), a systematic, planned and evidence-based strategy to promote positive social and behavioural changes.[24] It addresses the stigma, discrimination and exclusion of children with disability and forms an important part in making the Akwenda cerebral palsy programme accessible and acceptable. Target groups include community leaders, healthcare professionals, school educators, the general population, families, and the caregivers themselves.

Caregivers of the children with CP in the intervention group will participate in three C&A meetings to be conducted in months one, two, and six of the intervention. Each meeting will last 90 minutes and be led by the community social worker. It will take place on the same day as one of the parent-led workshops. Topics for these meetings are (i) rights of children with disabilities with a focus on human rights and abuses of children with CP in the community;

For peer review only

(ii) mapping available health, education and legal services in the community; and (iii) barriers to services and how to overcome those barriers. These discussions and the proposed solutions will then be discussed with the community leaders at a joint dialogue session the 7th month.

Forty community representatives, including political, administrative, cultural and religious leaders, educators, health workers, police, disability NGO/CBO representatives, district and sub-county technical personnel will be invited to participate in two C&A meetings led by the community social worker. The first session will focus on the rights of children with disabilities and the stigma they meet leading to discrimination and barriers limiting their participation. The second will be a joint session with the caregivers discussing how to make services inclusive and to develop a community action plan. The community social worker will follow up the implementation of the community action plan in months nine to eleven of the intervention through regular telephone feedback from participants and/or visits to the participating communities.

In preparation for the C & A component and to sensitize the community to disability issues, information on cerebral palsy will be delivered through a one-hour talk show on six community radio stations by the community social worker. Messages will include information relating to how the CURIE III study intends to assist children with cerebral palsy in Iganga-Mayuge. A telephone number of the community social worker will be shared with the listeners at the end of each radio talk show.

Time table and delivery of the integrated intervention

The time table for delivering the intervention through the five activities is shown in Figure 3. It will be delivered over a period of 11 months with caregivers attending two sessions per month, one of which will be together with the child. In addition, there will be three home visits and five C&A sessions. Two therapists, four parent facilitators and one community social worker will deliver the services required.

Discussion

The aim of the Akwenda CP intervention programme for children and youth with CP is to provide a comprehensive spectrum of interventions for the child, the caregiver and the community. The core of the intervention is a carer-to-carer training programme. Parent training programmes have previously been reported from Ghana and West Bengal, [7,8] however, that training was either delivered by health workers or community disability workers, only some of whom were mothers of children with disabilities.

In order to make this intervention holistic and comprehensive to impact the child, the caregiver and the wider community, the carer-to-carer training has been complemented with additional activities aiming to improve children's functional abilities, activities and participation as well as a community and advocacy programme aiming at social and behavioural changes in family and community. Good clinical practice should strive to address

For peer review only

the various ICF domains at the same time since improvements in one domain may not transfer directly to others.[25]

In line with WHO's goal of universal health coverage, we have tried to make the Akwenda CP programme physically accessible, affordable and acceptable. The venue for the intervention is a community setting within easy reach of the children and caregivers, making it physically accessible in a timely manner with minimal transportation costs. The intervention is designed to be affordable for caregivers living in a low resource setting where many are living at or below the poverty level, and with a scarcity of therapists and other rehabilitation professionals. Therapists are essential but their skills and time need to be optimised and leveraged, and the use of caregivers as in this programme, is one way to achieve this. TAD are largely locally manufactured.

Stigma and discrimination increases the risk that children with CP are neglected by primary health care and education services. The communication and advocacy component aims at social and behavioural change in the local community but particularly targeting health workers, teachers and community leaders. Empowerment of children and parents will increase their confidence and emphasize their rights to receive basic health and rehabilitation services. Working with caregivers and children in group settings, as opposed to working on an individual one to one setting, will also unify parents in similar situations and strengthen their confidence.

Currently, there are few evidence-based interventions that have been evaluated in LMICs. The activities of the Akwenda CP intervention programme is therefore based on best clinical practice derived from clinical studies in HIC, and adapted to the conditions in this rural part of Uganda in accordance with the WHO's goal of universal health coverage and universal access.[9] The multiple activities add to the complexity of the intervention as well as its evaluation. This study will provide information on the affordability and cost-benefit ratio of the provision of a comprehensive intervention in low-resource setting.

References:

1. Onward M. CARER-2-CARER TRAINING PROGRAMME 2020 [Available from: https://www.malamuleleonward.org/what-we-offer/carer-2-carer-programme.
2. Saloojee G, Bezuidenhout M. Parent-led services and community-based peer supporters for children and adults with disabilities: experiences from two programmes. In: Padarath A, Kathard H, Lorenzo T, et al., eds. South African Health Review 2020. Durban: Health Systems Trust 2020.
3. World Health Organization. International classification of functioning, disability and health: children and youth version: ICF-CY 2007 [Available from: https://apps.who.int/iris/handle/10665/43737.
4. King G, Williams L, Goldberg SH. Family-oriented services in pediatric rehabilitation: a scoping review and framework to promote parent and family wellness. *Child Care Hlth Dev* 2017;43(3):334-47. doi: 10.1111/cch.12435
5. Novak I, McIntyre S, Morgan C, et al. A systematic review of interventions for children with cerebral palsy: state of the evidence. *Dev Med Child Neurol* 2013;55(10):885-910. doi: 10.1111/dmcn.12246

6. Diaz Heijtz R, Forssberg H. Translational studies exploring neuroplasticity associated with motor skill learning and the regulatory role of the dopamine system. *Dev Med Child Neurol* 2015;57 Suppl 2:10-4. doi: 10.1111/dmcn.12692
7. Benfer KA, Novak I, Morgan C, et al. Community-based parent-delivered early detection and intervention programme for infants at high risk of cerebral palsy in a low-resource country (Learning through Everyday Activities with Parents (LEAP-CP): protocol for a randomised controlled trial. *BMJ Open* 2018;8(6):e021186. doi: 10.1136/bmjopen-2017-021186
8. Zuurmond M, Nyante G, Baltussen M, et al. A support programme for caregivers of children with disabilities in Ghana: Understanding the impact on the wellbeing of caregivers. *Child Care Health Dev* 2019;45(1):45-53. doi: 10.1111/cch.12618
9. Evans DB, Hsu J, Boerma T. Universal health coverage and universal access: World Health Organization; 2013 [Available from: http://dx.doi.org/10.2471/BLT.13.125450 [PDF].
10. Kakooza-Mwesige A, Andrews C, Peterson S, et al. Prevalence of cerebral palsy in Uganda: a population-based study. *Lancet Glob Health* 2017;5(12):e1275-e82. doi: 10.1016/S2214-109X(17)30374-1
11. Andrews C, Kakooza-Mwesige A, Almeida R, et al. Impairments, functional limitations, and access to services and education for children with cerebral palsy in Uganda: a population-based study. *Dev Med Child Neurol* 2019 doi: 10.1111/dmcn.14401
12. Kakooza-Mwesige A, Tumwine JK, Eliasson AC, et al. Malnutrition is common in Ugandan children with cerebral palsy, particularly those over the age of five and those who had neonatal complications. *Acta Paediatr* 2015;104(12):1259-68. doi: 10.1111/apa.13089
13. Andrews C, Kakooza-Mwesige A, Almeida R, et al. Impairments, functional limitations, and access to services and education for children with cerebral palsy in Uganda: a population-based study. *Dev Med Child Neurol* 2020;62(4):454-62. doi: 10.1111/dmcn.14401
14. WHO. Global Cooperation on Assistive Technology (GATE) 2020 [Available from: https://www.who.int/disabilities/technology/gate/en/.
15. Ketelaar M, Vermeer A, Hart H, et al. Effects of a functional therapy program on motor abilities of children with cerebral palsy. *Phys Ther* 2001;81(9):1534-45. doi: 10.1093/ptj/81.9.1534
16. Ahl LE, Johansson E, Granat T, et al. Functional therapy for children with cerebral palsy: an ecological approach. *Dev Med Child Neurol* 2005;47(9):613-9.
17. Bower E, McLellan DL, Arney J, et al. A randomised controlled trial of different intensities of physiotherapy and different goal-setting procedures in 44 children with cerebral palsy. *Dev Med Child Neurol* 1996;38(3):226-37. doi: 10.1111/j.1469-8749.1996.tb15084.x
18. Lowing K, Bexelius A, Brogren Carlberg E. Activity focused and goal directed therapy for children with cerebral palsy--do goals make a difference? *Disabil Rehabil* 2009;31(22):1808-16. doi: 10.1080/09638280902822278
19. Lowing K, Bexelius A, Carlberg EB. Goal-directed functional therapy: a longitudinal study on gross motor function in children with cerebral palsy. *Disabil Rehabil* 2010;32(11):908-16. doi: 10.3109/09638280903353422
20. Russell DJ, Avery LM, Rosenbaum PL, et al. Improved scaling of the gross motor function measure for children with cerebral palsy: evidence of reliability and validity. *Phys Ther* 2000;80(9):873-85.
21. Kakooza-Mwesige A, Tumwine JK, Forssberg H, et al. The Uganda version of the Pediatric Evaluation of Disability Inventory (PEDI). Part I: Cross-cultural adaptation.

For peer review only

Child Care Health Dev 2018;44(4):552-61. doi: 10.1111/cch.12563 [published Online First: 2018/03/13]
22. Amer A, Kakooza-Mwesige A, Jarl G, et al. The Ugandan version of the Pediatric Evaluation of Disability Inventory (PEDI-UG). Part II: Psychometric properties. *Child Care Health Dev* 2018;44(4):562-71. doi: 10.1111/cch.12562
23. Arvidsson P, Dada S, Granlund M, et al. Content validity and usefulness of Picture My Participation for measuring participation in children with and without intellectual disability in South Africa and Sweden. *Scand J Occup Ther* 2019:1-13. doi: 10.1080/11038128.2019.1645878
24. UNICEF. UNICEF 2017 Report on Communication for Development (C4D) 2017 [Available from: https://www.unicef.org/media/47781/file/UNICEF_2017_Report_on_Communication_for_Development_C4D.pdf.
25. Novak I, Morgan C, Fahey M, et al. State of the Evidence Traffic Lights 2019: Systematic Review of Interventions for Preventing and Treating Children with Cerebral Palsy. *Curr Neurol Neurosci Rep* 2020;20(2):3. doi: 10.1007/s11910-020-1022-z

For peer review only

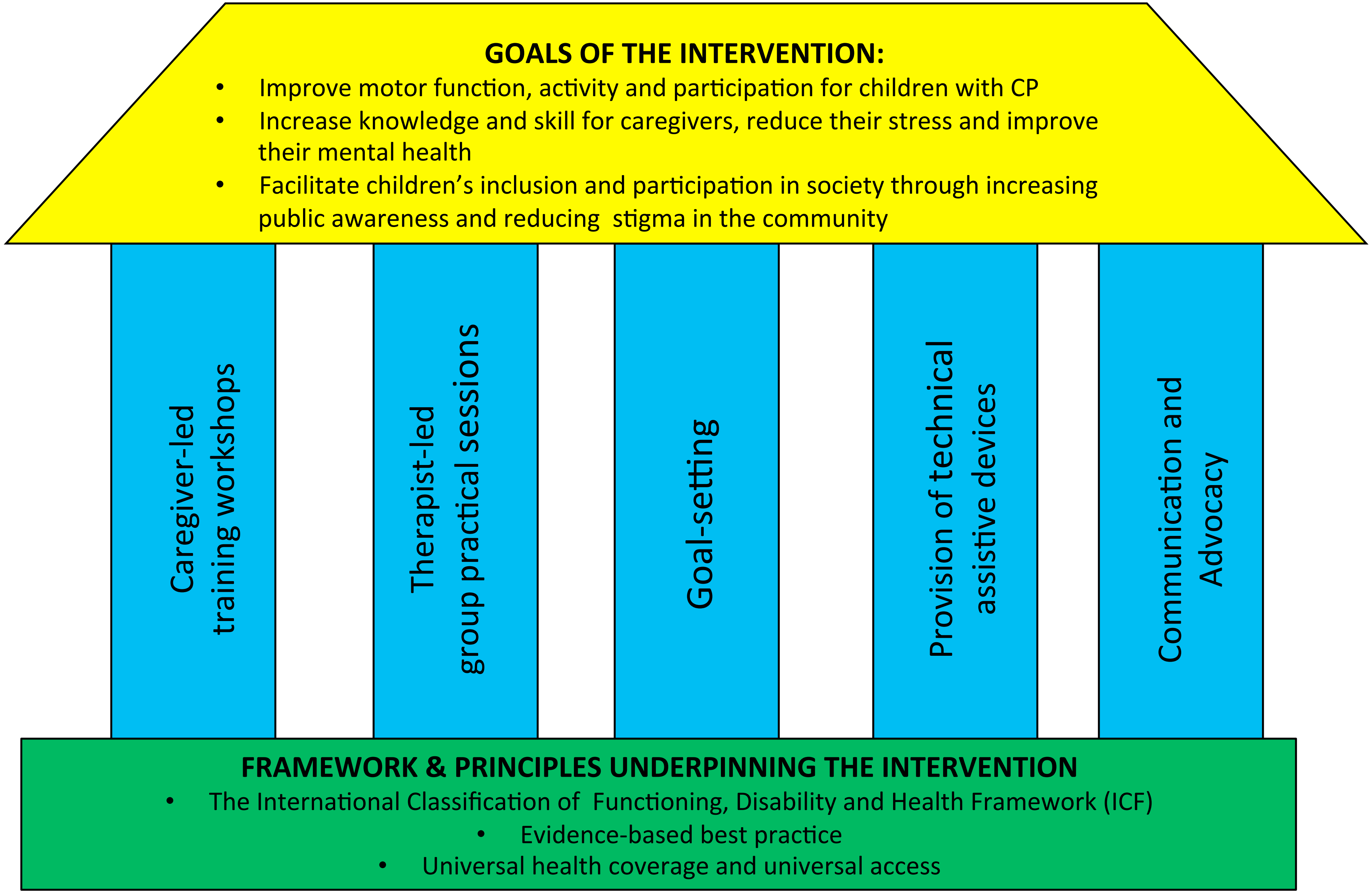
GOALS OF THE INTERVENTION:
Improve motor function, activity and participation for children with CP
Increase knowledge and skill for caregivers, reduce their stress and improve their mental health
Facilitate children's inclusion and participation in society through increasing public awareness and reducing stigma in the community
Caregiver-led training workshops
Therapist-led group practical sessions
Goal-setting
Provision of technical assistive devices
Communication and Advocacy
FRAMEWORK & PRINCIPLES UNDERPINNING THE INTERVENTION
The International Classification of Functioning, Disability and Health Framework (ICF)
Evidence-based best practice
Universal health coverage and universal access

SPIRIT

STANDARD PROTOCOL ITEMS: RECOMMENDATIONS FOR INTERVENTIONAL TRIALS

SPIRIT 2013 Checklist: Recommended items to address in a clinical trial protocol and related documents*

Section/item	Item No	Description	Addressed on page number
**Administrative information**			
Title	1	Descriptive title identifying the study design, population, interventions, and, if applicable, trial acronym	______1______
Trial registration	2a	Trial identifier and registry name. If not yet registered, name of intended registry	______2 & 11__
	2b	All items from the World Health Organization Trial Registration Data Set	______11______
Protocol version	3	Date and version identifier	______N/A_____
Funding	4	Sources and types of financial, material, and other support	_____13_______
Roles and responsibilities	5a	Names, affiliations, and roles of protocol contributors	____1 and 13___
	5b	Name and contact information for the trial sponsor	______N/A_____
	5c	Role of study sponsor and funders, if any, in study design; collection, management, analysis, and interpretation of data; writing of the report; and the decision to submit the report for publication, including whether they will have ultimate authority over any of these activities	______13/14__
	5d	Composition, roles, and responsibilities of the coordinating centre, steering committee, endpoint adjudication committee, data management team, and other individuals or groups overseeing the trial, if applicable (see Item 21a for data monitoring committee)	______N/A______

For peer review only

**Introduction**			
Background and rationale	6a	Description of research question and justification for undertaking the trial, including summary of relevant studies (published and unpublished) examining benefits and harms for each intervention	____4-5______
	6b	Explanation for choice of comparators	_____N/A_____
Objectives	7	Specific objectives or hypotheses	_____5-6_______
Trial design	8	Description of trial design including type of trial (eg, parallel group, crossover, factorial, single group), allocation ratio, and framework (eg, superiority, equivalence, noninferiority, exploratory)	_____6____
**Methods: Participants, interventions, and outcomes**			
Study setting	9	Description of study settings (eg, community clinic, academic hospital) and list of countries where data will be collected. Reference to where list of study sites can be obtained	______6____
Eligibility criteria	10	Inclusion and exclusion criteria for participants. If applicable, eligibility criteria for study centres and individuals who will perform the interventions (eg, surgeons, psychotherapists)	______6
Interventions	11a	Interventions for each group with sufficient detail to allow replication, including how and when they will be administered	7 - 8 + Suppl 1
	11b	Criteria for discontinuing or modifying allocated interventions for a given trial participant (eg, drug dose change in response to harms, participant request, or improving/worsening disease)	______N/A_____
	11c	Strategies to improve adherence to intervention protocols, and any procedures for monitoring adherence (eg, drug tablet return, laboratory tests)	_____Suppl 1_
	11d	Relevant concomitant care and interventions that are permitted or prohibited during the trial	______8_______
Outcomes	12	Primary, secondary, and other outcomes, including the specific measurement variable (eg, systolic blood pressure), analysis metric (eg, change from baseline, final value, time to event), method of aggregation (eg, median, proportion), and time point for each outcome. Explanation of the clinical relevance of chosen efficacy and harm outcomes is strongly recommended	_____9 - 10____
Participant timeline	13	Time schedule of enrolment, interventions (including any run-ins and washouts), assessments, and visits for participants. A schematic diagram is highly recommended (see Figure)	6, Fig 1,Table 1 + Suppl 1

For peer review only - http://bmjopen.bmj.com/site/about/guidelines.xhtml

Sample size	14	Estimated number of participants needed to achieve study objectives and how it was determined, including clinical and statistical assumptions supporting any sample size calculations	______8_____
Recruitment	15	Strategies for achieving adequate participant enrolment to reach target sample size	______8_____

Methods: Assignment of interventions (for controlled trials)

Allocation:

Sequence generation	16a	Method of generating the allocation sequence (eg, computer-generated random numbers), and list of any factors for stratification. To reduce predictability of a random sequence, details of any planned restriction (eg, blocking) should be provided in a separate document that is unavailable to those who enrol participants or assign interventions	______7_____
Allocation concealment mechanism	16b	Mechanism of implementing the allocation sequence (eg, central telephone; sequentially numbered, opaque, sealed envelopes), describing any steps to conceal the sequence until interventions are assigned	______7_______
Implementation	16c	Who will generate the allocation sequence, who will enrol participants, and who will assign participants to interventions	_______7_____
Blinding (masking)	17a	Who will be blinded after assignment to interventions (eg, trial participants, care providers, outcome assessors, data analysts), and how	_______7_____
	17b	If blinded, circumstances under which unblinding is permissible, and procedure for revealing a participant's allocated intervention during the trial	______N/A_____

Methods: Data collection, management, and analysis

Data collection methods	18a	Plans for assessment and collection of outcome, baseline, and other trial data, including any related processes to promote data quality (eg, duplicate measurements, training of assessors) and a description of study instruments (eg, questionnaires, laboratory tests) along with their reliability and validity, if known. Reference to where data collection forms can be found, if not in the protocol. Copies of the data collection forms can be obtained from the corresponding author.	_______9-10___
	18b	Plans to promote participant retention and complete follow-up, including list of any outcome data to be collected for participants who discontinue or deviate from intervention protocols	______N/A______

Data management	19	Plans for data entry, coding, security, and storage, including any related processes to promote data quality (eg, double data entry; range checks for data values). Reference to where details of data management procedures can be found, if not in the protocol	_____10-11_____
Statistical methods	20a	Statistical methods for analysing primary and secondary outcomes. Reference to where other details of the statistical analysis plan can be found, if not in the protocol	______11_______
	20b	Methods for any additional analyses (eg, subgroup and adjusted analyses)	______11_______
	20c	Definition of analysis population relating to protocol non-adherence (eg, as randomised analysis), and any statistical methods to handle missing data (eg, multiple imputation)	______N/A______
**Methods: Monitoring**			
Data monitoring	21a	Composition of data monitoring committee (DMC); summary of its role and reporting structure; statement of whether it is independent from the sponsor and competing interests; and reference to where further details about its charter can be found, if not in the protocol. Alternatively, an explanation of why a DMC is not needed	___11-12_______
	21b	Description of any interim analyses and stopping guidelines, including who will have access to these interim results and make the final decision to terminate the trial	______12_______
Harms	22	Plans for collecting, assessing, reporting, and managing solicited and spontaneously reported adverse events and other unintended effects of trial interventions or trial conduct	______12_______
Auditing	23	Frequency and procedures for auditing trial conduct, if any, and whether the process will be independent from investigators and the sponsor	______N/A_____
**Ethics and dissemination**			
Research ethics approval	24	Plans for seeking research ethics committee/institutional review board (REC/IRB) approval	______11_____
Protocol amendments	25	Plans for communicating important protocol modifications (eg, changes to eligibility criteria, outcomes, analyses) to relevant parties (eg, investigators, REC/IRBs, trial participants, trial registries, journals, regulators)	_______11__

Consent or assent	26a	Who will obtain informed consent or assent from potential trial participants or authorised surrogates, and how (see Item 32)	_____ 11____
	26b	Additional consent provisions for collection and use of participant data and biological specimens in ancillary studies, if applicable	______N/A___
Confidentiality	27	How personal information about potential and enrolled participants will be collected, shared, and maintained in order to protect confidentiality before, during, and after the trial	_____11_______
Declaration of interests	28	Financial and other competing interests for principal investigators for the overall trial and each study site	______14_____
Access to data	29	Statement of who will have access to the final trial dataset, and disclosure of contractual agreements that limit such access for investigators	_______14______
Ancillary and post-trial care	30	Provisions, if any, for ancillary and post-trial care, and for compensation to those who suffer harm from trial participation	_______N/A_____
Dissemination policy	31a	Plans for investigators and sponsor to communicate trial results to participants, healthcare professionals, the public, and other relevant groups (eg, via publication, reporting in results databases, or other data sharing arrangements), including any publication restrictions	_______12____
	31b	Authorship eligibility guidelines and any intended use of professional writers	______N/A______
	31c	Plans, if any, for granting public access to the full protocol, participant-level dataset, and statistical code	_______14______
**Appendices**			
Informed consent materials	32	Model consent form and other related documentation given to participants and authorised surrogates	____Attached ___
Biological specimens	33	Plans for collection, laboratory evaluation, and storage of biological specimens for genetic or molecular analysis in the current trial and for future use in ancillary studies, if applicable	_______N/A____

*It is strongly recommended that this checklist be read in conjunction with the SPIRIT 2013 Explanation & Elaboration for important clarification on the items. Amendments to the protocol should be tracked and dated. The SPIRIT checklist is copyrighted by the SPIRIT Group under the Creative Commons "Attribution-NonCommercial-NoDerivs 3.0 Unported" license.

For peer review only - http://bmjopen.bmj.com/site/about/guidelines.xhtml
